# Evaluating and establishing national norms for mental wellbeing using the short Warwick–Edinburgh Mental Well-being Scale (SWEMWBS): findings from the Health Survey for England

**DOI:** 10.1007/s11136-016-1454-8

**Published:** 2016-11-16

**Authors:** Linda Ng Fat, Shaun Scholes, Sadie Boniface, Jennifer Mindell, Sarah Stewart-Brown

**Affiliations:** 1 Department of Epidemiology and Public Health, University College London, 1-19 Torrington Place, London, WC1E 7HB UK; 2 Institute of Psychiatry, Psychology and Neuroscience, King’s College London, 4 Windsor Walk, Denmark Hill, London, SE5 8BB UK; 3 Division of Health Sciences, Warwick Medical School, University of Warwick, Coventry, CV4 7AL UK

**Keywords:** Mental wellbeing, Population norms, Instrument evaluation

## Abstract

**Purpose:**

The Warwick–Edinburgh Mental Well-being Scale (WEMWBS), 14 positively worded statements, is a validated instrument to measure mental wellbeing on a population level. Less is known about the population distribution of the shorter seven-item version (SWEMWBS) or its performance as an instrument to measure wellbeing.

**Methods:**

Using the Health Survey for England 2010–2013 (*n* = 27,169 adults aged 16+, nationally representative of the population), age- and sex-specific norms were estimated using means and percentiles. Criterion validity was examined using: (1) Spearman correlations (*ρ*) for SWEMWBS with General Health Questionnaire (GHQ-12), happiness index, EQ-VAS (2) a multinomial logit model with SWEMWBS (low, medium and high wellbeing) as the outcome and demographic, social and health behaviours as explanatory variables. Relative validity was examined by comparing SWEMWBS with WEMWBS using: (1) Spearman correlations (continuous data), and (2) the weighted kappa statistic (categorical), within population subgroups.

**Results:**

Mean (median) SWEMWBS was 23.7 (23.2) for men and 23.2 (23.2) for women (*p* = 0.100). Spearman correlations were moderately sized for the happiness index (*ρ* = 0.53, *P* < 0.001), GHQ-12 (*ρ* = −0.52, *p* < 0.001) and EQ-VAS (*ρ* = 0.40, *p* < 0.001). Participants consuming <1 portion of fruit and vegetables a day *versus* ≥5 (odds ratio = 1.43 95% Confidence Interval = (1.22–1.66)) and current smokers *versus* non-smokers (1.28 (1.15–1.41)) were more likely to have low *vs* medium wellbeing. Participants who binge drank *versus* non-drinkers were less likely to have high versus medium wellbeing (0.81 (0.71–0.92)). Spearman correlations between SWEMWBS and WEMWBS were above 0.95; weighted kappa statistics showed almost perfect agreement (0.79–0.85).

**Conclusion:**

SWEMWBS distinguishes mental wellbeing between subgroups, similarly to WEMWBS, but is less sensitive to gender differences.

**Electronic supplementary material:**

The online version of this article (doi:10.1007/s11136-016-1454-8) contains supplementary material, which is available to authorized users.

## Background

There has been growing interest in measuring mental wellbeing, recognising that mental health is more than the absence of mental illness, and the desire for policy makers to assess progress outside of the usual economic and material indicators [1–3]. Mental wellbeing has been defined by different authorities as various combinations of optimum functioning and feeling [4]. Mental wellbeing has been found to have a U-shaped relationship with age [5, 6]. It is linked with good physical health and with longevity among older adults [7]. Its relationship with social and economic circumstances is complex [6, 8].

The Warwick–Edinburgh Mental Wellbeing Scale (WEMWBS) was developed in 2007 [9] to support the development of an evidence base relating to public mental health. Public mental health encompasses the promotion of mental wellbeing, the prevention of mental illness and recovery from mental illness. The 14 items of the WEMWBS scale are all positively worded and relate to the main components of mental wellbeing, defined as ‘feeling good and functioning well’ [4]. Its strengths include the ability to capture both the eudaimonic (people’s functioning, social relationships, sense of purpose) and the hedonic perspectives on wellbeing (e.g. feelings of happiness). In most validation studies, scores resolve to a single component.

In 2009, a short version (seven items) of the scale (SWEMWBS) was resolved using the Rasch measurement model [10]. Five items were removed from the 14-item WEMWBS to improve the overall fit of the data to the Rasch model; and two items were removed due to local item dependency (i.e. residual associations in the data after the Rasch-based trait score had been removed). The remaining seven-item scale fitted the expectations of the Rasch model, and a linear transformation of the score was then obtained, to facilitate the use of valid parametric statistical analyses. The items in SWEMWBS present a picture of mental wellbeing in which psychological functioning dominates subjective feeling states, but the superior scaling properties and reduced participant burden have made it the instrument of choice in some studies. Both scales have proved very popular with practitioners and researchers in the UK and further afield. There were 1841 registrations for use between October 2012 to March 2016, and the numbers are increasing annually [11].

Although both scales have been used to evaluate interventions and to examine the epidemiology of mental wellbeing, more research has been published on the full 14-item scale, including population norms for European countries [12–15]. A recent study in England [8] showed surprising findings relating to the social distribution of mental wellbeing. The expected social inequalities distribution was demonstrated for those at the lower end of the mental wellbeing scale—a group at high risk of mental health problems—but not for those at the high end of the mental wellbeing scale. Differences between predictors of the low end of the wellbeing scale with the high end of the wellbeing scale were also found with health behaviours. Obesity and being a non-drinker of alcohol were associated with the low end of the mental wellbeing scale but not with the high end, while smoking and low fruit and vegetable intake were associated with both increased odds of the low and decreased odds of the high end of the scale [16]. Whether the short seven-item scale exhibits similar properties to the full 14-item scale in a nationally representative sample in England has yet to be explored. SWEMWBS may have lower face validity than the full scale, focusing on items relating to functioning and excluding items relating to feeling aspects [17]. Since the short scale is being used widely in England, it is important to establish national norms for the short scale and evaluate how it performs against the full scale, so that practitioners and researchers using SWEMWBS, for example those conducting small-scale studies on local areas, have a meaningful benchmark with which to compare their results. This study therefore aimed to compare the performance of SWEMWBS and WEMWBS in the English population.

## Methods

### Aims of the study

We aimed to answer the following research questions:

1. What are the national norms for SWEMWBS in the general population in England and across socio-demographic subgroups? How do subgroup differences in scores on SWEMWBS compare with those on WEMWBS?
2. How well does SWEMWBS correlate with GHQ-12, EQ-VAS, happiness index, and self-reported health and limiting longstanding illness, as compared to correlation of WEMWBS with such instruments?
3. Does SWEMWBS reproduce associations with social and health behaviour variables similar to the full version?
4. How closely does the measurement of mental wellbeing with SWEMWBS approximate to the measurement by WEMWBS, and within different subgroups? In addition, how well does SWEMWBS capture those at the low and high ends of the mental wellbeing scale compared with WEMWBS?

### Study participants

The study uses data from the Health Survey for England 2010–2013 (*N* = 27,169), the first survey years which included the 14-item WEMWBS [18–21]. The Health Survey for England interviews each year a new, random, nationally representative sample of the population living in private households in England [22]. Participants were selected using a multi-stage, stratified, probability design, with postcode sectors used as primary sampling units, randomly selected using the Postcode Address file. Data included spoken answers to questions, written answers in self-completion booklets, and biomedical information, which was collected via face-to-face interviews followed by a nurse visit. WEMWBS was self-completed confidentially as part of the interviewer visit, with the exception of 2012 when this was done during the nurse visit. NHS Research Ethics Committee approval was obtained prior to each survey commencing from the Oxford B (2010) and Oxford A (2011–13) Research Ethics Committees. Participants gave informed verbal consent prior to the interview.

### Data

#### WEMWBS and SWEMWBS

Answers to each item on WEMWBS (and SWEMWBS) are provided using a five-point Likert scale (none of the time, rarely, some of the time, often, all of the time), and scored from 1 to 5 respectively, with all items being scored positively. Scores on all items are then summed to give a WEMWBS score (range 14–70) (see Box 1).

SWEMWBS uses seven items from the full 14-item WEMWBS (items in bold in Box 1). As with WEMWBS, scores on SWEMWBS are summed (range 7–35). As described earlier, SWEMWBS scores were transformed (set out in a conversion table published in a previous study [10, 23]) to facilitate the use of parametric statistical analyses. SWEMWBS was embedded within the full scale, so each HSE participant had scores on both scales (with the exception of 512 participants who completed the seven SWEMWBS items but did not complete the full 14-item scale).

To examine whether SWEMWBS was able to capture those with lower wellbeing scores as well as WEMWBS, three-category versions of SWEMWBS and WEMWBS scores were derived. Low and high categories were based on scores that were at least one standard deviation below and above the mean, respectively [16]. Categories for SWEMWBS were: ‘low’: 7–19.3; ‘medium’: 20.0–27.0; and ‘high’: 28.1–35. For WEMWBS, scores were ‘low’: 14–42; ‘medium’: 43–60; and ‘high’: 61–70.

#### Demographic, socio-economic, health and health behaviour data

Data on sex, age group, marital status, ethnicity, highest educational qualification, quintiles of equivalised household income, economic status, self-rated health and limiting longstanding illness were reported in the face-to-face interview. Region and area-deprivation (derived from the Index of Multiple Deprivation) were based on the participant’s address.

Instruments measuring mental and overall health in the HSE included the General Health Questionnaire (GHQ-12), an instrument comprising scores from 12 questions measuring psychological morbidity (2010 and 2012 only). For each of the 12 questions, participants were given a four-point response scale, ranging from ‘not at all present’ to ‘present much more than usual’. The first two responses were coded as zero, and the third and fourth responses were coded as one, providing a maximum score of 12. In addition the EQ-VAS score, a visual analogue scale where participants rate their health from ‘worst imaginable health state’ (0) to ‘best imaginable health state’ (100) (2010–2012 only), and a happiness index (2010 and 2011 only) were included in the analysis. Within the happiness index, participants were asked to rate how happy they were from 0 (unhappy) to 10 (happy). These measures were collected via the same self-completion booklet that contained WEMWBS.

Health behaviours included current smoking status; alcohol consumption; and fruit and vegetable portions per day (not asked in 2012), which were self-reported. Body mass index categories were derived from height and weight measurements carried out by trained interviewers. Categorisation of alcohol consumption on the heaviest drinking day in the last 7 days was based on daily limits of alcohol consumption as recommended at the time of the survey (≤4 units a day for men, ≤3 units a day for women). These were as follows: non-drinker, moderate drinker (within daily limits), excess drinker (exceeding daily limits but less than twice the recommended limits) and heavy episodic drinker (over twice the recommended limits). Categorisation of fruit and vegetable consumption was as follows: 5 or more portions of fruit and vegetables a day, 3 to <5, 1 to <3, and <1 portion a day. BMI groups were defined as underweight (<18.5 kg/m^2^), normal weight (18.5 to <25 kg/m^2^), overweight (25 to <30 kg/m), obese (30 to <40 kg/m^2^) and morbidly obese (≥40 kg/m^2^). Physical activity was covered only in 2012, so numbers did not allow its inclusion in this study.

### Statistical analysis

#### Establishing Norms (research question 1)

Sex-stratified national norms for SWEMWBS were calculated, including the mean, 10, 15, 50, 85 and 90th centile across the key demographic variables. The same norms stratified by age group are shown in supplementary tables. Norms for the present study can be read along age, sex and one other dimension only.

First, we used univariable linear regression to estimate the difference in mean SWEMWBS scores fitting variables such as age group and income as categorical variables. Statistical significance was examined using a joint hypothesis test (i.e. whether the coefficients for the difference in mean scores were simultaneously equal to zero). Second, categorical variables such as income were fitted as continuous terms to estimate the change in SWEMWBS per unit change in the predictor. Third, the magnitude of the association was estimated with the effect size (ES), computed as the difference between the mean wellbeing scores of two subgroups, divided by the pooled standard deviation. Uncertainty in ES was estimated using bootstrap confidence intervals based on the noncentral t distribution. The cut-offs and the interpretation of ES were: low (|0.20| ≥ ES ≤ |0.50|), moderate (|0.50| > ES ≤ |0.80|) and high (ES > |0.80|). The same analyses were repeated for WEMWBS. We hypothesised that SWEMWBS would show similar variation across subgroups as WEMWBS.

#### Criterion validity (research question 2)

Spearman correlation coefficients (*ρ*) were estimated between SWEMWBS and the five variables of physical and mental health including GHQ-12 score, EQ-VAS, happiness index, self-rated health and limiting longstanding illness. To account for the complex survey design (including non-response weighting), the rank of the variable was regressed on the rank of SWEMWBS. Since the Spearman correlation coefficient is equal to the slope of the regression between the ranked values of the two measures, its value was estimated by regressing the rank of participants on SWEMWBS on the rank of the physical and mental health variable [24]. In the present study, SWEMWBS was embedded in WEMWBS, and to avoid the issue of overlap, we also randomly split the data into two halves (*N*_1_ = 13,584, *N*_2_ = 13,311) and carried out the same analyses on the two independent samples for SWEMWBS (N_1_) and WEMWBS (N_2_), respectively. This is presented in the supplementary tables. In addition, to examine the internal consistency of the shorter scale as compared with WEMWBS, we calculated Cronbach’s alpha for each scale, with a value of over 0.70 considered to be indicative of acceptable internal reliability [25].

We expected correlations between physical and mental health variables and SWEMWBS to be of a similar magnitude to correlations with WEMWBS. In line with the literature on WEMWBS, we hypothesised that SWEMWBS would have statistically significant but moderate correlations with GHQ-12 [9] and lower correlations with variables that measure overall health, such as EQ-VAS, the former measuring mental ill health and the latter measuring overall health, which are different from mental wellbeing.

#### Similarities in association with social and health variables (research question 3)

To address research aim three, the three-category versions of SWEMWBS and WEMWBS were used as outcome variables in separate multinomial logistic regression models, comparing low with medium wellbeing and high with medium wellbeing. The decision to model SWEMWBS as a categorical variable rather than continuous was based on the different associations at the low and high end of the spectrum found in a previous study [8]. Modelling SWEMWBS as a continuous variable therefore would mean that some of these differing properties may be masked. Variables in single, fully adjusted models included sex, age group, marital status, ethnic group, highest educational qualification, economic status, equivalised income quintiles, self-rated general health, body mass index, fruit and vegetable intake, alcohol consumption, smoking status and survey year. To maximise all available cases on each variable, missing data were recoded into a ‘missing’ category, including missing 2012 data on fruit and vegetable consumption. However, we also repeated the analysis using listwise deletion which is presented in the supplementary tables. We prefer to present the former as the main model as it maximised all available information, including data from 2012.

#### Relative validity (research question 4)

To assess the extent of agreement between the two scales, we used the Bland–Altman method to plot the difference in scores for each respondent (WEMWBS–SWEMWBS) against the mean of the two scores. WEMWBS score was first divided by two to make the scale comparable to SWEMWBS, which ranges from 7 to 35. The Bland–Altman plot enables a visual inspection of the association between the differences in scores and the magnitude of wellbeing. Spearman correlation coefficients were calculated between SWEMWBS and WEMWBS, both overall and within subgroups, to explore similarities in the consistency of rankings. Since SWEMWBS was embedded in WEMWBS, potentially leading to upward bias in the estimates of correlation, we also present Spearman correlation coefficients between SWEMWBS and the seven items from the 14-item WEMWBS that were not included in the shorter scale. To explore the classification accuracy of SWEMWBS relative to WEMWBS, weighted kappa statistics were calculated between the three-category version of SWEMWBS and WEMWBS, and repeated within population subgroups. To assess the strength of agreement, we used the Landis and Koch classification [26]: slight: 0–0.20; fair: 0.21–0.40; moderate: 0.41–0.60; substantial: 0.61–0.80; and almost perfect: 0.81–1.00. Percentage agreement in the classification was also assessed.

Non-response weighting (which accounts for non-response by households, individuals within co-operating households and, for HSE 2012, non-response to the nurse visit) was applied to all analyses. Data management was performed using SPSS version 20.0 (SPSS Inc., Chicago, Illinois, US) and analysis was conducted using Stata version 14 (StataCorp LP, College Station, Texas, US) accounting for the complex sample design.

## Results

Around 80% of the original sample (*N* = 34,155) answered all seven SWEMWBS items (*N* = 27,169), which was around 2% higher than the number of participants who answered the 14 item WEMWBS (*N* = 26,617). The response rate by year corresponded to 85% in 2010, 84% in 2011 and 61% in 2012 (information collected during the nurse visit), and 88% in 2013 within co-operating households.

## Norms

Tables 1 (men) and 2 (women) present national norms for SWEMWBS across social and demographic variables, with *p* values for the joint hypothesis test (i.e. whether the coefficients for the difference in mean scores across categories were simultaneously equal to zero) and *p*-values for the null hypothesis of zero change in SWEMWBS per unit change in the continuous predictor. The same analyses were carried out for WEMWBS (presented in supplementary Table 1). In addition, norms for socio-economic, demographic and health sub-categories by age group are presented for SWEMWBS in supplementary Tables 2 (men) and 3 (women).

**Table 1:** SWEMWBS scores for men across social and demographic groups, HSE 2010–2013^a^

	*N*	Mean	SD	10th	15th	50th	85th	90th	Effect size (ES)	*p* value	*p* value for one-unit change
All men	11,948	23.67	3.92	19.25	19.98	23.21	27.03	28.13			
Age group											
16–24	1143	23.57	3.61	19.25	19.98	23.21	27.03	28.13	Reference		
25–34	2366	23.53	3.57	19.25	19.98	23.21	27.03	28.13	−0.01 (−0.08, 0.07)		
35–44	1998	23.43	3.86	18.59	19.25	23.21	27.03	28.13	−0.03 (−0.10, 0.04)		
45–54	2107	23.45	3.99	18.59	19.25	23.21	27.03	28.13	−0.03 (−0.10, 0.04)		
55–64	2054	23.82	4.03	19.25	19.98	24.11	27.03	28.13	0.06 (−0.02, 0.13)		
65–74	1821	24.52	4.19	19.25	20.73	24.11	28.13	29.31	0.25 (0.17, 0.33)		
75+	1208	23.82	4.41	29.31	23.67	19.25	19.98	23.21	0.08 (−0.01–0.16)	*p* < 0.001	*p* < 0.001
Index of multiple deprivation											
Least	2653	24.08	3.75	19.25	20.73	24.11	28.13	29.31	Reference		
2nd	2588	23.83	3.66	19.25	19.98	24.11	27.03	28.13	−0.08 (−0.14, −0.02)		
3rd	2534	23.63	3.89	18.59	19.98	23.21	27.03	28.13	−0.12 (−0.17, −0.06)		
4th	2204	23.65	4.02	19.25	19.25	23.21	27.03	28.13	−0.15 (−0.21, −0.09)		
Most	1969	23.08	4.25	17.98	18.59	23.21	27.03	28.13	−0.30 (−0.36, −0.23)	*p* < 0.001	*p* < 0.001
Education											
Degree or higher	3098	24.31	3.58	19.98	20.73	24.11	28.13	29.31	Reference		
Below degree	6473	23.55	3.85	19.25	19.98	23.21	27.03	28.13	−0.20 (−0.24, −0.20)		
No qualification	2361	23.10	4.46	17.98	18.59	23.21	27.03	28.13	−0.22 (−0.26, −0.18)	*p* < 0.001	*p* < 0.001
Income quintiles											
Highest	2321	24.30	3.57	19.98	20.73	24.11	27.03	29.31	Reference		
2nd	2306	23.97	3.54	19.98	20.73	24.11	27.03	28.13	−0.09 (−0.15, −0.04)		
3rd	2011	23.76	3.77	19.25	19.98	23.21	27.03	28.13	−0.14 (−0.20, −0.08)		
4th	1800	23.39	4.11	18.59	19.25	23.21	27.03	28.13	−0.24 (−0.30, −0.17)		
Lowest	1486	22.59	4.36	17.43	17.98	22.35	27.03	28.13	−0.45 (−0.52, −0.38)	*p* < 0.001	*p* < 0.001
Region											
North East	1001	23.40	4.09	18.59	19.25	23.21	27.03	28.13	Reference		
North West	1633	23.48	4.10	18.59	19.25	23.21	27.03	28.13	0.01 (−0.07, 0.8)		
Yorkshire and The Humber	1150	23.51	4.00	18.59	19.25	23.21	27.03	28.13	0.03 (−0.05, 0.11)		
East Midlands	1119	23.74	3.90	19.25	19.98	24.11	27.03	28.13	0.09 (0.01, 0.17)		
West Midlands	1228	23.41	3.94	18.59	19.25	23.21	27.03	28.13	−0.00 (−0.09, 0.08)		
East of England	1336	23.74	3.80	19.25	19.98	23.21	27.03	28.13	0.09 (0.00, 0.17)		
London	1274	23.97	4.02	19.25	19.98	24.11	28.13	29.31	0.13 (0.04, 0.21)		
South East	1939	23.89	3.73	19.25	19.98	24.11	27.03	28.13	0.13 (0.05, 0.21)		
South West	1268	23.61	3.74	19.25	19.98	23.21	27.03	28.13	0.08 (−0.01, 0.17)	*p* = 0.0018	n/a
Ethnicity											
White	10,829	23.59	3.87	18.59	19.98	23.21	27.03	28.13	Reference		
Mixed	119	24.04	3.80	19.25	19.98	24.11	28.13	29.31	0.05 (0.11, −0.20)		
Asian	652	24.08	4.16	19.25	19.98	24.11	28.13	29.31	0.15 (0.05, 0.21)		
Black	260	25.24	4.33	19.98	20.73	25.03	30.70	30.70	0.37 (0.24, 0.49)		
Other	70	24.22	4.37	17.98	19.25	24.11	28.13	28.13	0.15 (0.11, 0.41)	*p* < 0.001	n/a
Self-rated health											
Very good	3948	24.87	3.66	20.73	21.54	25.03	28.13	29.31	Reference		
Good	5098	23.66	3.62	19.25	19.98	23.21	27.03	28.13	−0.32 (−0.36, −0.36)		
Fair	2072	22.39	3.94	17.98	18.59	22.35	26.02	27.03	−0.67 (−0.072, −0.61)		
Bad	634	20.24	4.03	15.84	16.36	19.98	24.11	25.03	−1.25 (−1.34, −1.15)		
Very bad	195	19.32	4.08	14.75	15.84	18.59	23.21	24.11	−1.52 (−1.69, −1.35)	*p* < 0.001	*p* < 0.001
Limiting longstanding illness											
None	6828	24.08	4.24	19.98	20.73	24.11	27.03	28.13	Reference		
Longstanding illness	2391	24.13	3.91	19.25	20.73	24.11	28.13	29.31	−0.02 (−0.03, −0.06)		
Limiting longstanding illness	2723	22.00	3.69	16.88	17.98	21.54	26.02	27.03	−0.54 (−0.60, −0.49)	*p* < 0.001	*p* < 0.001

^a^Sex differences for SWEMWBS (*p* = 0.100, ES = 0.03; 95% CI: (0.01–0.06))

**Table 2:** SWEMWBS scores for women across social and demographic groups, HSE 2010–13

	*N*	Mean	SD	10th	15th	50th	85th	90th	Effect size (ES)	*p* value	*p* value for one-unit change
All women	15,221	23.59	3.99	18.59	19.25	23.21	27.03	28.13			
Age group											
16–24	1540	23.17	3.86	18.59	19.25	23.21	27.03	28.13	Reference		
25–34	2282	23.68	3.80	19.25	19.98	24.11	27.03	28.13	0.13 (0.07, 0.19)		
35–44	2682	23.46	3.89	18.59	19.25	23.21	27.03	28.13	0.09 (0.03, 0.14)		
45–54	2840	23.31	3.88	18.59	19.25	23.21	27.03	28.13	0.04 (−0.02, 0.11)		
55–64	2431	23.95	4.10	19.25	19.98	24.11	28.13	29.31	0.21 (0.15, 0.27)		
65–74	1964	24.26	4.31	19.25	19.98	24.11	28.13	30.70	0.29 (0.22, 0.35)		
75+	1482	23.59	4.23	18.59	19.25	23.21	28.13	23.21	0.13 (0.06, 0.20)	*p* < 0.001	*p* < 0.001
Index of multiple deprivation											
Least	3348	23.95	3.81	19.25	19.98	24.11	27.03	29.31	Reference		
2nd	3256	23.87	3.83	19.25	19.98	24.11	27.03	28.13	−0.04 (−0.08, 0.01)		
3rd	3198	23.69	4.01	19.25	19.98	23.21	27.03	29.31	−0.08 (−0.13, −0.04)		
4th	2828	23.37	3.96	18.59	19.25	23.21	27.03	28.13	−0.19 (−0.24, −0.14)		
Most	2591	22.93	4.32	17.98	18.59	23.21	27.03	28.13	−0.28 (−0.33, −0.23)	*p* < 0.001	*p* < 0.001
Education											
Degree or higher	3607	24.26	3.62	19.98	20.73	24.11	28.13	29.31	Reference		
Below degree	8352	23.46	3.96	18.59	19.25	23.21	27.03	28.13	−0.19 (−0.22, −0.15)		
No qualification	3245	23.15	4.40	17.98	18.59	23.21	27.03	29.31	−0.15 (−0.19, −0.10)	*p* < 0.001	*p* < 0.001
Income quintiles											
Highest	2478	24.35	3.69	19.98	20.73	24.11	28.13	29.31	Reference		
2nd	2701	24.02	3.66	19.98	20.73	24.11	27.03	28.13	−0.08 (−0.14, −0.2)		
3rd	2512	23.64	3.88	19.25	19.98	23.21	27.03	28.13	−0.18 (−0.23, 0.12)		
4th	2497	23.24	4.06	18.59	19.25	23.21	27.03	28.13	−0.28 (−0.33, −0.22)		
Lowest	2276	22.59	4.37	17.43	18.59	22.35	27.03	28.13	−0.45 (−0.51, −0.39)	*p* < 0.001	*p* < 0.001
Region											
North East	1324	23.03	3.93	17.98	19.25	23.21	27.03	27.03	Reference		
North West	1982	23.58	4.17	18.59	19.25	23.21	27.03	29.31	0.12 (0.05, 0.20)		
Yorkshire and The Humber	1480	23.46	4.10	18.59	19.25	23.21	27.03	28.13	0.11 (0.03, 0.18)		
East Midlands	1440	23.45	3.97	18.59	19.25	23.21	27.03	28.13	0.10 (0.20, 0.17)		
West Midlands	1504	23.33	3.94	18.59	19.25	23.21	27.03	28.13	0.07 (−0.0, 0.15)		
East of England	1672	23.70	4.02	19.25	19.98	23.21	27.03	28.13	0.17 (0.10, 0.25)		
London	1706	23.79	4.00	19.25	19.98	23.21	28.13	29.31	0.18 (0.10, 0.25)		
South East	2517	23.81	3.91	19.25	19.98	24.11	27.03	28.13	0.21 (0.14, 0.27)		
South West	1596	23.67	3.83	19.25	19.98	23.21	27.03	28.13	0.16 (0.08, 0.24)	*p* < 0.001	n/a
Ethnicity											
White	13,778	23.56	3.94	18.59	19.98	23.21	27.03	28.13	Reference		
Mixed	165	23.43	4.15	18.59	19.25	23.21	28.13	28.13	−0.06 (−0.22,0.10)		
Asian	784	23.92	4.36	18.59	19.25	24.11	28.13	29.31	0.05 (−0.02, 0.13)		
Black	361	24.12	4.39	18.59	19.98	24.11	29.31	29.31	0.17 (0.05, 0.29)		
Other	102	23.38	4.34	18.59	18.59	23.21	27.03	29.31	−0.04 (−0.25, 0.18)	*p* = 0.1037	n/a
Self-rated health											
Very good	5021	24.88	3.80	19.98	21.54	25.03	28.13	29.31	Reference		
Good	6457	23.66	3.67	19.25	19.98	23.21	27.03	28.13	−0.32 (−0.36, −0.29)		
Fair	2782	22.08	3.87	17.98	18.59	21.54	26.02	27.03	−0.71 (−0.76, −0.67)		
Bad	722	20.34	3.92	15.84	16.88	19.98	24.11	25.03	−1.19 (−1.28, −1.11)		
Very bad	236	19.59	4.73	15.32	15.32	19.25	24.11	25.03	−1.42 (−1.56, −1.27)	*p* < 0.001	*p* < 0.001
Limiting longstanding illness											
None	8653	24.09	4.18	19.25	19.98	24.11	28.13	29.31	Reference		
Longstanding illness	2700	24.02	3.80	19.25	19.98	24.11	28.13	29.31	−0.02 (−0.06, −0.02)		
Limiting longstanding illness	3859	22.05	3.81	17.43	17.98	21.54	26.02	27.03	−0.52 (−0.56, −0.48)	*p* < 0.001	*p* < 0.001

Mean SWEMWBS scores for men and women were 23.7 and 23.6, respectively (ES = 0.03, 95% CI: 0.01–0.06), and were not statistically different (*p* = 0.100). The largest differences across mean scores of SWEMWBS were observed across the categories of self-rated health, ranging from 19.3 for men reporting ‘very bad’ health to 24.7 for men reporting ‘very good’ health (ES = −1.52), and 19.6–24.9 for women (ES = −1.42). Effect sizes for limiting longstanding illness (versus none) were moderate in magnitude (ES = −0.54 and −0.52 for men and women, respectively). Mean scores for SWEMWBS varied significantly across the categories of income, education and Index of Multiple Deprivation (*p* < 0.05). Effect sizes for the lowest income quintile (versus highest) ranged from small to moderate. With regard to age, the largest effect size was observed for the 65–74 group versus the 16–24 group (ES = 0.25 and 0.29 for men and women, respectively). Differences in mean SWEMWBS scores across the nine Government Office Regions were statistically different to zero (*p* < 0.001), but the effect sizes were small in magnitude (ES < |0.20|). Differences in SWEMWBS scores across ethnic groups were statistically significant for men but not for women; the effect size for Black men (vs. White men) was moderate in magnitude (ES = 0.37).

Variation in scores on WEMWBS by age and across subgroups followed a similar pattern to SWEMWBS (supplementary Table 1), including the magnitude of effect sizes. However, in contrast to SWEMWBS, gender differences in wellbeing scores using the 14-item scale were statistically significant (*p* = 0.009), but the estimated change in wellbeing score for a one-unit change in age group (fitted as a continuous term) was not significantly different from zero (*p* = 0.749).

## Criterion validity

Table 3 presents Spearman correlations between mental and physical health variables and both SWEMWBS and WEMWBS. Statistically significant but moderate correlations between SWEMWBS and the happiness index (*ρ* = 0.53, *p* < 0.001), GHQ12 (*ρ* = −0.52, *p* < 0.001) and EQ-VAS (*ρ* = 0.40, *p* < 0.001) were found. There were weaker correlations with self-rated health (*ρ* = −0.33, *p* < 0.001) and limiting longstanding illness (*ρ* = −0.21, *p* < 0.001).

**Table 3:** Spearman correlation coefficient between SWEMWBS/WEMWBS and health variables, HSE 2010–13

	SWEMWBS		WEMWBS	
	*N*	*ρ*	*N*	*ρ*
Self-rated health	27,165	−0.33***	26,613	−0.36***
Limiting longstanding illness	27,154	−0.21***	26,602	−0.23***
GHQ12^a^	11,688	−0.52***	11,386	−0.52***
Happiness scale^b^	12,952	0.53***	12,661	0.56***
EQ-VAS Scale^c^	17,978	0.40***	17,559	0.42***

*** *p* < 0.001

^a^HSE 2010 and 2012 only

^b^HSE 2010 and 2011 only

^c^EQ-VAS: Visual analogue Scale. HSE 2010–2012 only

Correlation coefficients were very similar between SWEMWBS and WEMWBS; where they differed, WEMWBS had slightly higher correlations (up to 0.03 difference). Correlations with the mental and physical health variables were of a similar magnitude for SWEMWBS and WEMWBS even when comparing across the two different, randomly generated samples (Supplementary table S4). With regard to the internal reliability of the scales, Cronbach’s alpha for SWEMWBS and WEMWBS was 0.84 and 0.92, respectively, both exceeding the acceptable conventional level of internal agreement (0.70).

Table 4 presents results from multinomial logistic regressions for SWEMWBS categorised into low (15%), medium (71%) and high (14%) wellbeing (proportions were the same for WEMWBS to zero decimal points). Focusing on SWEMWBS scores only, and the low versus the medium wellbeing categories, participants aged 25–54 were more likely to have low than medium wellbeing compared with 16- to 24-year-olds. Participants with worse self-rated health were more likely to have low than medium wellbeing (e.g. bad/very bad health: odds ratio (OR) = 9.51 (95 % confidence interval 8.05–11.23)). Similar gradients were demonstrated for education (e.g. no qualifications OR = 1.42 (1.23–1.65)) and income (e.g. lowest quintile 1.48 (1.26–1.73)). Eating less than one portion of fruit and vegetables a day compared with five or more was associated with an increased odds of low versus medium wellbeing (1.42 (1.22–1.67)). Current smokers were more likely than non-smokers to have low versus medium wellbeing (1.28 (1.15–1.41)). Moderate drinkers were less likely than non-drinkers to have low wellbeing (0.87 (0.79–0.97)). Associations with low versus medium wellbeing as measured by WEMWBS showed a similar overall pattern to SWEMWBS, although there were a few differences in which comparisons attained statistical significance, e.g. participants in the Black ethnic group having the lowest odds of low wellbeing (vs. White participants) on SWEMWBS (0.68 (0.51–0.92)), and participants in the mixed ethnic group having the lowest odds of low wellbeing on WEMWBS (0.56 (0.36–0.87)).

**Table 4:** Multinomial logistic regression comparing low versus medium, high versus medium wellbeing measured by SWEMWBS and WEMWBS, HSE 2010–2013^a,b^

Variables (reference category)	Low versus medium		High versus medium	
	SWEMWBS	WEMWBS	SWEMWBS	WEMWBS
	OR (95% CI)	OR (95% CI)	OR (95% CI)	OR (95% CI)
Sex (Men)	1		1	
Women	1.01 (0.94–1.09)	1.06 (0.98–1.15)	1.00 (0.93–1.08)	0.97 (0.90–1.05)
Age group (16–24)				
25–34	**1.25 (1.05–1.50)**	**1.30 (1.08–1.56)**	0.89 (0.72–1.10)	0.85 (0.70–1.05)
35–44	**1.47 (1.23–1.76)**	**1.63 (1.35–1.96)**	1.03 (0.83–1.27)	0.96 (0.78–1.18)
45–54	**1.30 (1.08–1.57)**	**1.57 (1.30–1.91)**	1.12 (0.91–1.39)	1.07 (0.87–1.32)
55–64	0.93 (0.76–1.14)	1.06 (0.86–1.31)	**1.58 (1.27–1.98)**	**1.45 (1.16–1.80)**
65–74	**0.77 (0.59–0.99)**	0.80 (0.61–1.04)	**2.10 (1.63–2.70)**	**1.90 (1.49–2.43)**
75+	0.86 (0.66–1.13)	0.87 (0.66–1.15)	**2.09 (1.59–2.75)**	**1.88 (1.44–2.44)**
General health (very good)				
Good	**1.85 (1.65–2.08)**	**1.95 (1.73–2.20)**	**0.54 (0.50–0.59)**	**0.53 (0.48–0.58)**
Fair	**4.33 (3.78–4.95)**	**4.80 (4.16–5.54)**	**0.35 (0.31–0.40)**	**0.31 (0.27–0.36)**
Bad/very bad	**9.51 (8.05–11.23)**	**11.86 (10.00–14.06)**	**0.21 (0.16–0.28)**	**0.19 (0.14–0.26)**
Marital status (single)	1		1	
Married/cohabitees	**0.69 (0.61–0.77)**	**0.63 (0.55–0.71)**	**1.15 (1.00–1.32)**	1.11 (0.97–1.27)
Separated/widowed/divorced	0.88 (0.75–1.02)	0.87 (0.75–1.01)	1.01 (0.86–1.20)	0.95 (0.80–1.12)
Ethnic group (White)	1		1	
Mixed	0.81 (0.55–1.19)	**0.56 (0.36–0.87)**	1.39 (0.98–1.99)	1.31 (0.91–1.89)
Asian	0.83 (0.69–1.01)	0.82 (0.67–1.00)	**1.56 (1.28–1.91)**	**1.48 (1.21–1.81)**
Black	**0.68 (0.51–0.92)**	0.77 (0.57–1.04)	**2.25 (1.77–2.87)**	**2.25 (1.73–2.93)**
Other	1.09 (0.69–1.72)	1.04 (0.67–1.62)	1.26 (0.76–2.10)	1.55 (0.88–2.72)
Education (degree or higher)	1		1	
Below degree	**1.23 (1.10–1.37)**	**1.23 (1.09–1.38)**	0.95 (0.86–1.06)	0.95 (0.86–1.04)
No qualification	**1.42 (1.23–1.64)**	**1.38 (1.19–1.60)**	0.99 (0.86–1.14)	0.98 (0.85–1.12)
Economic activity (in employment)	1		1	
ILO unemployed	**1.34 (1.12–1.61)**	**1.32 (1.09–1.59)**	0.92 (0.74–1.16)	1.10 (0.89–1.36)
Retired	1.00 (0.84–1.19)	0.99 (0.83–1.18)	**1.17 (1.01–1.34)**	**1.21 (1.04–1.40)**
Other economically inactive	**1.51 (1.34–1.71)**	**1.52 (1.34–1.72)**	1.11 (0.96–1.29)	**1.17 (1.01–1.36)**
Equivalised income quintile (highest)	1		1	
2nd	0.98 (0.84–1.15)	0.96 (0.82–1.13)	0.89 (0.78–1.01)	**0.87 (0.77–0.98)**
3rd	1.15 (0.99–1.33)	1.08 (0.92–1.26)	0.89 (0.77–1.02)	0.87 (0.76–1.00)
4th	**1.31 (1.11–1.54)**	**1.30 (1.10–1.53)**	0.90 (0.77–1.05)	0.91 (0.78–1.06)
Lowest	**1.48 (1.26–1.73)**	**1.49 (1.26–1.76)**	0.88 (0.74–1.03)	0.92 (0.77–1.09)
Body mass index (normal)	1		1	
Underweight	1.14 (0.83–1.57)	1.14 (0.82–1.60)	0.99 (0.68–1.44)	0.90 (0.60–1.35)
Overweight	0.97 (0.88–1.08)	1.01 (0.91–1.13)	1.07 (0.96–1.18)	0.97 (0.88–1.08)
Obese	0.91 (0.80–1.02)	0.95 (0.84–1.08)	**1.22 (1.09–1.37)**	**1.14 (1.01–1.28)**
Morbidly obese	1.07 (0.85–1.34)	1.16 (0.92–1.47)	**1.66 (1.29–2.13)**	1.24 (0.94–1.63)
Fruit and Vegetable intake (5 or more portions/day)	1		1	
3 to <5 portions/day	0.99 (0.88–1.12)	1.01 (0.90–1.15)	**0.85 (0.76–0.95)**	**0.87 (0.78–0.97)**
1 to <3 portions/day	**1.18 (1.05–1.33)**	**1.22 (1.08–1.37)**	**0.78 (0.69–0.89)**	**0.73 (0.64–0.82)**
<1 portions/day	**1.43 (1.22–1.67)**	**1.54 (1.31–1.81)**	**0.76 (0.63–0.93)**	**0.76 (0.63–0.93)**
Alcohol drinking (non-drinker)	1		1	
Moderate	**0.87 (0.79–0.97)**	**0.82 (0.74–0.92)**	**0.85 (0.77–0.94)**	**0.86 (0.77–0.95)**
Excess	0.93 (0.82–1.06)	0.88 (0.77–1.00)	**0.84 (0.74–0.95)**	0.90 (0.80–1.02)
Heavy episodic	0.92 (0.81–1.04)	0.90 (0.79–1.02)	**0.81 (0.72–0.93)**	**0.84 (0.73–0.96)**
Smoking (never smoker)	1		1	
Ex-smoker	1.09 (0.99–1.21)	1.10 (1.00–1.22)	0.94 (0.86–1.03)	0.92 (0.84–1.01)
Current smoker	**1.28 (1.15–1.41)**	**1.26 (1.13–1.40)**	1.07 (0.94–1.21)	0.99 (0.87–1.13)

^a^Adjusted for all variables shown in the table, and survey year

^b^Figures in bold are statistically significant at the 5% level (*p* < 0.05)

For the high versus the medium wellbeing categories for SWEMWBS, older age groups (aged 55+) were more likely to have high wellbeing than 16–24-year-olds. This is in contrast to the finding of the younger age groups having higher odds of low wellbeing and demonstrates the well-known U-shaped association between wellbeing and age. Those with worse self-rated health were also the least likely to have high wellbeing (e.g. bad/very bad health OR = 0.21 (0.16–0.28)). However, the categories of income and educational status showed no association with the odds of high wellbeing, unlike the findings for the odds of low versus medium wellbeing. Participants in the Asian (OR = 1.56 (1.28–1.91) and Black ethnic groups (OR = 2.25 (1.77–2.87) were more likely to have high wellbeing than participants in the White ethnic group. There were gradients in the associations with lower fruit and vegetable consumption, and with higher alcohol consumption, with lower odds of high versus medium wellbeing found for participants in these groups (e.g. <1 portion a day (0.76 (0.63–0.93); >8 units alcohol 0.81 (0.72–0.93)). Obese (1.22 (1.09–1.37)) and morbidly obese (1.66 (1.29–2.13)) participants were more likely to have high wellbeing than those with normal weight; although overweight and obese participants had higher odds of having low versus medium wellbeing when adjustment excluded self-reported health (see Discussion). Again, analyses using WEMWBS showed a similar overall pattern, but some categories differed in whether they attained statistical significance: for example, associations for marital status and morbid obesity were not statistically significant for WEMWBS. Models using the subset of participants with complete data (supplementary table S5) showed no substantial differences between SWEMWBS and WEMWBS, nor with the main models.

## Relative validity

**Fig. 1:**
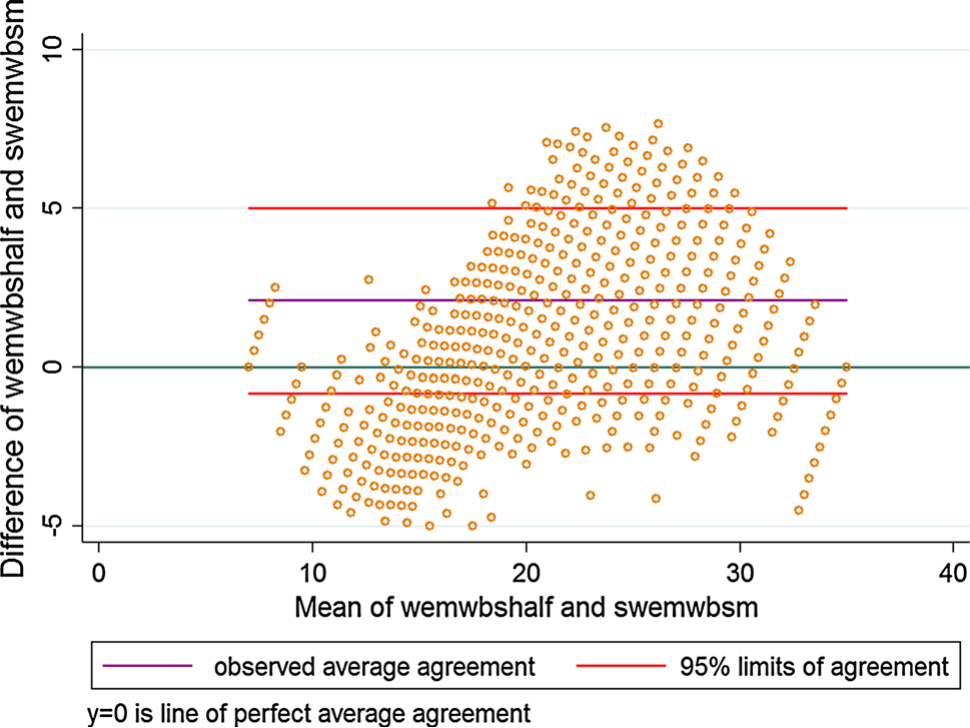
Bland-Altman Plot of the difference between WEMWBS and SWEMWBS scores against the the mean of WEMWBS and SWEMWBS

The Bland–Altman plot for the comparison of each instrument is depicted in Fig. 1. The average discrepancy between the SWEMWBS and WEMWBS scores was 2.1 (95% CI: −0.80–5.01). The difference in scores demonstrated proportional error, with a slight tendency for this to increase with larger mean scores. The line of equality fell within the 95% CI of the mean difference meaning no absolute bias. Table 5 presents Spearman correlations between SWEMWBS and WEMWBS, and weighted kappa statistics between SWEMWBS and WEMWBS grouped into low, medium and high categories, within different subgroups. Correlations between SWEMWBS and WEMWBS were very high and statistically significant (0.95–0.96, *p* < 0.001) within subgroups of sex, education, income and the Index of Multiple Deprivation. For self-rated health, correlations were high and statistically significant, although slightly lower in magnitude (0.80–0.85, *p* < 0.001). Coefficients were also high, albeit lower in magnitude, for the comparisons of SWEMWBS with the seven redundant items in WEMWBS (0.84–0.87, *p* < 0.001). Weighted kappa coefficients showed substantial to almost perfect relative agreement between the two classifications across subgroups (0.79–0.85).

**Table 5:** Spearman correlation, percentage agreement and weighted kappa coefficient between SWEMWBS and WEMWBS, within demographic subgroups

	SWEMWBS correlation with WEMWBS		SWEMWBS correlation with 7 items from WEMWBS not included	SWEMWBS and WEMWBS categories		
				Weighted kappa		
	*N*	*ρ*		Agreement %	Kappa coefficient	95% CI
All	26,617	0.95***	0.85***	97.22	0.843	(0.84–0.85)
Sex						
Men	11,707	0.95***	0.85***	97.69	0.839	(0.83–0.85)
Women	14,910	0.95***	0.85***	97.75	0.846	(0.84–0.86)
Age group						
16–24	2650	0.95***	0.83***	97.42	0.816	(0.79–0.84)
25–34	3837	0.94***	0.83***	97.97	0.839	(0.82–0.85)
35–44	4616	0.95***	0.86***	97.95	0.852	(0.84–0.87)
45–54	4861	0.96***	0.87***	97.80	0.847	(0.84–0.86)
55–64	4399	0.96***	0.87***	97.84	0.854	(0.81–0.87)
65–74	3680	0.95***	0.85***	97.50	0.835	(0.82–0.85)
75+	2574	0.95***	0.84***	97.24	0.836	(0.08–0.85)
Index of multiple deprivation						
Least	5922	0.95***	0.85***	98.05	0.850	(0.84–0.86)
2nd	5727	0.95***	0.84***	97.66	0.823	(0.81–0.84)
3rd	5623	0.95***	0.85***	97.73	0.843	(0.83–0.86)
4th	4923	0.95***	0.85***	97.56	0.835	(0.82–0.85)
Most	4422	0.96***	0.87***	97.54	0.856	(0.84–0.87)
Education						
Degree or higher	6624	0.95***	0.84***	98.00	0.833	(0.82–0.84)
Other	14,574	0.95***	0.86***	97.74	0.842	(0.83–0.85)
None	5386	0.95***	0.85***	97.34	0.847	(0.84–0.86)
Income quintiles						
Highest	4749	0.95***	0.84***	92.50	0.846	(0.82–0.86)
2nd	4945	0.95***	0.84***	91.79	0.826	(0.81–0.84)
3rd	4446	0.95***	0.84***	90.78	0.831	(0.81–0.85)
4th	4193	0.95***	0.85***	90.25	0.845	(0.83–0.86)
Lowest	3645	0.96***	0.86***	90.21	0.861	(0.84–0.88)
General health						
Very good	8842	0.81***	0.83***	97.79	0.821	(0.81–0.83)
Good	11,326	0.80***	0.88***	97.84	0.826	(0.82–0.84)
Fair	4720	0.81***	0.83***	97.44	0.834	(0.82–0.85)
Bad/very bad	1725	0.85***	0.87***	97.33	0.837	(0.81–0.86)

*** Significant at the < 0.001 level

**Box 1:** 14 items on the WEMWBS scale, with the seven items of SWEMWBS highlighted in bold

*I’ve been feeling optimistic about the future*
***I’ve been feeling useful***
***I’ve been feeling relaxed***
*I’ve been feeling interested in other people*
*I’ve had energy to spare*
***I’ve been dealing with problems well***
***I’ve been thinking clearly***
*I’ve been feeling good about myself*
***I’ve been feeling close to other people***
*I’ve been feeling confident*
***I’ve been able to make up my own mind about things***
*I’ve been feeling loved*
*I’ve been interested in new things*
*I’ve been feeling cheerful*

## Discussion

SWEMWBS performed comparably to WEMWBS in these analyses, demonstrating the expected population distributions and correlations with social variables for low wellbeing, and mimicking recent findings demonstrated with WEMWBS for high wellbeing. There was proportional disagreement presented in the Bland–Altman plot, reflecting the difference in scaling for SWEMWBS transformed to a metric scale, while no such transformation was required for WEMWBS. This small difference between the scales could also have affected differences found between SWEMWBS and WEMWBS in other analyses. However, despite this, SWEMWBS behaved very similarly to WEMWBS. The well-documented income and education gradients for low versus medium wellbeing were not found for high versus medium wellbeing. Similar moderate correlations were found between SWEMWBS and GHQ12 and EQ-VAS, as had been previously demonstrated for WEMWBS [9]. In men, SWEMWBS also followed the well-known U-shaped distribution by age for wellbeing, with its nadir between 35 and 55 years for low wellbeing [5, 9]. In women, we observed a slight difference in the norms for the two scales as its nadir was in the 16–24 age group, making the U-shaped distribution by age less clear.

The main difference between the performances of the two measures related to gender. Norms for WEMWBS were slightly higher for men, whereas for SWEMWBS norms did not vary significantly by gender. This is consistent with a study that found SWEMWBS to be gender neutral in a Swedish and Norweigan population [27]. The items common to both instruments include feeling useful, dealing with problems well, thinking clearly and autonomy. The majority of the seven WEMWBS items that are not present in the SWEMWBS relate more to the affective or feelings components of wellbeing (feeling good about self, confident, cheerful, loved, having energy to spare): each of which varied significantly by gender (*p* < 0.001, data not shown). It is therefore not surprising that WEMWBS detects more gender differences than SWEMWBS. The other two WEMWBS items not present in SWEMWBS relate to functioning (interest in new things; feeling interested in other people), which did not vary significantly by gender (*p* = 0.126 and *p* = 0.776, respectively, data not shown) [12]. However, it is important to note that average scores on WEMWBS may not vary much by gender, given by results in other contexts [12] and the small effect sizes found in this study. Surprising results relating to high versus medium wellbeing included the increased odds in Black and Asian ethnic groups, and in those who were obese, found with both instruments. Increased odds of high wellbeing among ethnic minority groups have been found before [6, 8], in particular among the Black minority ethnic group, which was suggested to be driven largely by high mean scores for wellbeing among Black African groups [6]. Black African groups were also found to have better self-reported health than White British groups after extensive adjustment for health behaviour and SEP confounders [28]; this may be attributable to a ‘healthy migrant effect’. Further studies wishing to look at ethnic differences in mental wellbeing should differentiate between Black Africans and Black Caribbeans and other heterogenous groups, where numbers allow. The minor differences between WEMWBS and SWEMWBS in the magnitude of the correlations observed for different ethnic groups are likely to be due to small sample sizes in some minority groups.

The higher odds of high versus medium mental wellbeing among overweight or obese participants were more marked with SWEMWBS than with WEMWBS and remain largely unexplained. It is important to recognise that these are only seen after adjustment for general health. Adjustment for general health in our models explains the different findings between health behaviours and wellbeing from those of Stranges et al. [16], including the non-significant associations between decreased odds of high wellbeing among non-smokers, and increased odds of low wellbeing among obese individuals.

The strong association between both low versus medium and high versus medium wellbeing and fruit and vegetable intake, even after adjustment for a number of socio-economic factors, suggests fruit and vegetable consumption as a possible causal factor in mental wellbeing. However, this present study was conducted using cross-sectional data and so we cannot rule out the possibility of reverse causality. Our findings must also be interpreted with caution due to the inevitable problem of residual confounding. Nevertheless, the associations between fruit and vegetable consumption and wellbeing deserve further investigation using longitudinal data. Since our primary aim was to evaluate how SWEMWBS performed against WEMWBS, further investigation was beyond the scope of the present study. It is also important that future studies examine the sensitivity to change of SWEMWBS compared with WEMWBS. Given the larger number of items in total, and the greater contribution of ‘feelings’ items, it remains possible that WEMWBS is more sensitive than SWEMWBS to change in intervention studies. This difference may prove important in small-scale evaluations of community-based mental wellbeing interventions [29].

## Limitations of this study

The participants who answered SWEMWBS in our sample were given the full WEMWBS questionnaire. Participants may respond differently if asked only the SWEMWBS subset of questions, due to different question ordering, the shorter length and the absence of any influence that the omitted questions in SWEMWBS may have on the full WEMWBS responses. Around 80% of the sample answered the SWEMWBS questionnaire. Among non-responders there was a higher proportion of males, those living in the most deprived quintile, and low qualifications than responders (*p* < 0.001, data not shown). It is likely that these people may have lower mental wellbeing; therefore, the norms for SWEMWBS shown in the present study may be overestimated. However, we feel that the use of a nationally representative survey, and the use of non-response weighting, offset this limitation. The consistency of our findings with other studies suggests that our results do not have large biases although we accept this limitation as a caveat to our findings. Our analysis has largely focused on a comparison of SWEMWBS with WEMWBS to evaluate SWEMWBS as a tool to measure mental wellbeing; however, we acknowledge that SWEMWBS is subject to the same limitations as WEMWBS; for instance, we found minimal effect sizes across certain subgroups such as region with both instruments, despite significant *p* values, which is likely to be an artefact of the large sample size. Measuring mental wellbeing as a single construct may mask its multidimensionality [30].

## Conclusions

SWEMWBS’s performance is very similar to that of WEMWBS. In this context, the 2% higher response rate observed for the SWEMWBS items within the Health Survey for England WEMWBS questionnaire, and its lower participant burden, will continue to make it a popular choice for both large-scale social surveys and intervention studies. However, those particularly interested in gender differences in mental wellbeing may prefer to use the full 14-item instrument. Further studies are needed to ensure that SWEMWBS performs as well as WEMWBS in terms of responsiveness to change in intervention studies.

## Availability of data and materials

The datasets supporting the conclusions of this article are available via the UK Data Service repository, subject to their end user license agreement:

NatCen Social Research, Royal Free and University College Medical School. Department of Epidemiology and Public Health. (2015). *Health Survey for England, 2010*. [data collection]. *3rd Edition.* UK Data Service. SN: 6986, http://dx.doi.org/10.5255/UKDA-SN-6986-3.

NatCen Social Research, University College London. Department of Epidemiology and Public Health. (2013). *Health Survey for England, 2011*. [data collection]. UK Data Service. SN: 7260, http://dx.doi.org/10.5255/UKDA-SN-7260-1.

NatCen Social Research, University College London. Department of Epidemiology and Public Health. (2014). *Health Survey for England, 2012*. [data collection]. UK Data Service. SN: 7480, http://dx.doi.org/10.5255/UKDA-SN-7480-1.

NatCen Social Research, University College London. Department of Epidemiology and Public Health. (2015). *Health Survey for England, 2013*. [data collection]. UK Data Service. SN: 7649, http://dx.doi.org/10.5255/UKDA-SN-7649-1.

## Electronic supplementary material

Below is the link to the electronic supplementary material.

Supplementary material 1 (PDF 1270 kb)
