# Supplementary material for: Evaluating and establishing national norms for mental wellbeing using the short Warwick–Edinburgh Mental Well-being Scale (SWEMWBS): findings from the Health Survey for England
Source: Qual Life Res. 2016 Nov 16;26(5):1129–44. doi: 10.1007/s11136-016-1454-8 (PMC5376387; doi:10.1007/s11136-016-1454-8)
Supplement: Supplementary file 1 — Supplementary material 1 (PDF 1270 kb) [file 11136_2016_1454_MOESM1_ESM.pdf]

Table S1 WEMWBS scores for men amd women across social and demographic groups, HSE 2010-13

Men

	N	Mean	s.d	10th	15th	50th	85th	90th	p-value	p-value for one unit change
All	11707	51.7	8.7	41	43	53	60	62		
**Age group**										
16-24	1131	52.4	8.1	42	44	53	60	62		
25-34	1596	51.9	7.9	42	44	53	59	61		
35-44	1973	51.2	8.7	40	42	52	60	62		
45-54	2063	50.8	9.2	39	42	52	60	62		
55-64	2011	51.7	9.0	40	42	53	60	62		
65-74	1780	53.2	8.8	42	45	54	62	64		
75+	1153	51.7	9.4	40	43	52	61	63	p<0.001	p=0.749
**Index of Multiple Deprivation**										
Least	2617	52.6	7.9	42	45	53	60	63		
2nd	2536	52.2	8.0	42	44	53	60	62		
3rd	2487	51.6	8.6	41	43	53	60	62		
4th	2155	51.8	8.9	41	43	53	61	62		
Most	1912	50.3	10.0	37	40	52	60	62	p<0.001	p<0.001
**Education**										
High	3064	53.4	7.5	43	46	54	61	63		
Other	6357	51.5	8.6	41	43	52	60	62		
None	2270	50.0	10.2	37	40	51	60	62	p<0.001	p<0.001
**Income quintiles**										
Highest	2297	53.3	7.5	44	46	54	61	62		
2nd	2276	52.5	7.6	43	45	53	60	62		
3rd	1974	51.9	8.2	42	43	52	60	62		
4th	1754	51.0	9.1	39	42	52	60	62		
Lowest	1441	48.9	10.5	35	38	50	59	61		
Missing	1965	51.6	9.1	40	42	53	61	63	p<0.001	p<0.001
**Region**										
North East	974	51.0	9.4	39	41	52	60	62		
North West	1606	51.3	9.2	39	42	52	60	62		
Yorkshire and	1126	51.2	9.1	40	43	52	60	62		
East Midlands	1104	51.9	8.4	42	44	53	60	62		
West Midlands	1189	51.0	9.0	39	42	52	60	62		
East of England	1304	51.9	8.3	41	43	53	60	62		
London	1252	52.5	8.7	42	44	54	61	63		
South East	1911	52.3	8.1	42	44	53	60	62		
South West	1241	51.7	8.3	41	43	53	60	62	p<0.001	n/a
**Ethnicity**										
White	10616	51.5	8.7	41	43	52	60	62		
Mixed	117	53.0	7.6	43	45	53	61	63		
Asian	635	52.9	8.8	42	44	54	62	64		
Black	252	55.0	8.8	43	46	55	65	67		
Other	69	52.9	10.0	38	40	55	64	64	p<0.001	n/a
**Self-rated health**										
Very good	3894	54.7	7.3	45	48	55	62	64		
Good	5003	51.9	7.7	42	44	52	60	62		
Fair	2008	48.2	9.2	37	39	49	57	60		
Bad	611	42.1	10.4	28	31	42	54	56		
Very bad	190	39.2	10.7	26	27	39	51	53	p<0.001	p<0.001
**Limiting longstanding illness**										
None	6721	53.0	7.7	43	45	53	61	63		
Longstanding	2336	52.7	8.0	42	45	53	60	62		
Limiting	2644	47.0	10.3	33	36	48	57	59	p<0.001	p<0.001

sex differences p=0.0088

Women

	N	Mean	s.d	10th	15th	50th	85th	90th	p-value	p-value for one unit change
All	14910	51.6	8.8	40	43	52	60	62		
**Age group**										
16-24	1519	51.0	8.7	39	42	52	60	62		
25-34	2241	51.9	8.4	41	43	53	60	62		
35-44	2643	51.1	8.8	40	42	52	59	61		
45-54	2798	50.7	9.0	39	42	52	59	61		
55-64	2388	52.1	8.9	41	43	53	61	63		
65-74	1900	52.7	9.0	42	44	53	62	64		
75+	1421	51.1	9.2	40	42	51	61	63	p<0.001	p=0.014
**Index of Multiple Deprivation**										
Least	3305	52.4	8.4	42	44	53	61	63		
2nd	3191	52.2	8.2	42	44	53	60	62		
3rd	3136	51.6	8.7	40	43	53	60	62		
4th	2768	51.0	8.9	40	42	52	60	62		
Most	2510	49.6	10.0	37	40	51	60	62	p<0.001	p<0.001
**Education**										
High	3560	53.3	7.7	43	46	54	61	63		
Other	8217	51.1	8.8	40	42	52	60	62		
None	3116	50.0	9.8	37	40	51	60	62	p<0.001	p<0.001
**Income**										
Highest	2452	53.5	7.7	43	46	54	61	63		
2nd	2669	52.6	7.8	43	45	53	60	62		
3rd	2472	51.7	8.3	41	43	52	60	62		
4th	2439	50.4	9.2	39	41	51	59	62		
Lowest	2204	48.7	10.2	35	38	50	58	61		
Missing	2674	51.4	9.1	40	42	52	60	63	p<0.001	p<0.001
**Region**										
North East	1294	50.1	9.1	39	41	51	59	60		
North West	1951	51.2	9.2	39	42	52	60	62		
Yorkshire and	1451	51.1	9.2	39	42	52	60	62		
East Midlands	1403	50.9	9.0	39	42	52	60	62		
West Midlands	1473	50.9	8.8	39	42	51	60	61		
East of	1640	51.6	8.8	40	43	52	60	62		
London	1660	52.1	8.7	41	43	53	61	63		
South East	2478	52.1	8.5	41	44	53	60	63		
South West	1560	51.6	8.4	41	43	52	60	62	p<0.001	n/a
**Ethnicity**										
White	13517	51.4	8.8	40	42	52	60	62		
Mixed	164	51.3	9.6	39	43	53	61	62		
Asian	753	52.3	9.3	40	42	54	61	63		
Black	350	52.4	9.7	39	42	53	62	64		
Other	97	51.4	9.0	42	42	52	61	63	p=0.111	n/a
**Self-rated**										
Very good	4948	54.7	7.6	45	47	55	62	64		
Good	6323	51.7	7.9	42	44	52	59	61		
Fair	2712	47.6	9.0	36	39	48	57	59		
Bad	698	42.6	10.2	29	32	43	53	55		
Very bad	226	40.7	11.5	26	28	41	53	55	p<0.001	p<0.001
**Limiting**										
None	8492	52.8	9.9	42	45	53	61	63		
Longstanding	2651	52.5	8.1	42	44	53	61	63		
Limiting	3758	47.3	8.0	35	37	48	57	59	p<0.001	p<0.001

S2. SWEMWBS scores for men by age group

		Index of Multiple Deprivation					Income quintiles				
	ALL	Least	2nd	3rd	4th	Most	Highest	2nd	3rd	4th	Lowest
**16-24**											
N	1143	228	205	230	209	271	123	174	185	158	242
Mean	23.57	23.86	23.69	23.13	24.20	23.11	23.38	23.90	23.41	24.05	23.15
s.d	3.61	3.69	3.33	3.64	3.36	3.83	3.91	3.69	3.10	3.74	3.86
10th centile	19.25	19.25	19.25	18.59	19.98	18.59	18.59	19.25	19.25	19.25	18.59
15th centile	19.98	19.98	19.98	19.25	20.73	19.25	19.98	20.73	19.98	19.98	19.25
50th centile	23.21	24.11	23.21	23.21	24.11	23.21	23.21	24.11	23.21	24.11	23.21
85th centile	27.03	27.03	27.03	26.02	27.03	27.03	27.03	27.03	27.03	27.03	27.03
90th centile	28.13	28.13	28.13	27.03	28.13	27.03	29.31	28.13	28.13	28.13	28.13
**25-34**											
N	2366	248	273	360	374	362	585	502	374	315	282
Mean	23.53	23.67	23.66	23.45	23.66	23.27	24.29	23.63	23.43	22.79	22.29
s.d	3.57	3.27	3.51	3.48	3.71	3.74	3.40	3.00	3.22	3.64	4.00
10th centile	19.25	19.98	19.98	18.59	19.25	18.59	20.73	19.98	19.25	18.59	17.43
15th centile	19.98	19.98	20.73	19.98	19.25	19.25	21.54	20.73	19.98	19.25	17.98
50th centile	23.21	24.11	23.21	23.21	24.11	23.21	24.11	23.21	23.21	23.21	21.54
85th centile	27.03	27.03	27.03	27.03	27.03	26.02	27.03	27.03	26.02	26.02	26.02
90th centile	28.13	28.13	28.13	28.13	28.13	27.03	28.13	27.03	27.03	27.03	27.03
**35-44**											
N	1998	430	419	408	392	349	504	448	324	259	221
Mean	23.43	23.91	23.44	23.63	22.97	23.21	24.19	23.76	23.57	22.94	21.83
s.d	3.86	3.52	3.50	3.97	4.10	4.15	3.34	3.54	3.76	3.76	4.33
10th centile	18.59	19.25	19.25	18.59	17.98	17.98	19.98	19.25	19.25	17.98	16.36
15th centile	19.25	19.98	19.98	19.25	19.25	18.59	20.73	19.98	19.98	19.25	16.88
50th centile	23.21	24.11	23.21	24.11	23.21	23.21	24.11	24.11	23.21	23.21	22.35
85th centile	27.03	27.03	27.03	28.13	26.02	27.03	27.03	27.03	27.03	26.02	26.02
90th centile	28.13	29.31	27.03	28.13	28.13	28.13	28.13	28.13	28.13	28.13	27.03
**45-54**											
N	2107	501	444	426	395	341	527	494	322	237	251
Mean	23.45	23.77	23.63	23.54	23.67	22.45	24.15	23.86	23.18	22.92	21.94
s.d	3.99	3.76	3.63	3.69	4.23	4.66	3.42	3.69	3.80	4.22	4.65
10th centile	18.59	19.25	19.25	19.25	18.59	16.88	19.98	19.25	18.59	17.98	16.36
15th centile	19.25	19.98	19.98	19.98	19.25	17.98	20.73	19.98	19.25	18.59	17.43
50th centile	23.21	24.11	23.21	23.21	24.11	22.35	24.11	24.11	23.21	22.35	22.35
85th centile	27.03	27.03	27.03	27.03	27.03	27.03	27.03	27.03	27.03	27.03	26.02
90th centile	28.13	28.13	28.13	28.13	28.13	28.13	28.13	28.13	28.13	28.13	27.03
**55-64**											
N	2054	503	464	432	359	296	458	440	328	232	268
Mean	23.82	24.27	24.38	23.58	23.48	22.93	24.74	24.16	24.28	22.92	22.11
s.d	4.03	3.72	3.80	3.87	4.00	4.90	3.79	3.43	4.02	4.26	4.46
10th centile	19.25	19.98	19.98	18.59	19.25	16.88	19.98	19.98	19.25	17.98	16.88
15th centile	19.98	20.73	20.73	19.25	19.25	18.59	20.73	20.73	19.98	18.59	17.98
50th centile	24.11	24.11	24.11	23.21	23.21	22.35	24.11	24.11	24.11	23.21	22.35
85th centile	27.03	28.13	28.13	27.03	27.03	27.03	28.13	27.03	28.13	27.03	26.02
90th centile	28.13	29.31	29.31	28.13	28.13	29.31	29.31	28.13	29.31	28.13	27.03
**65-74**											
N	1821	451	460	409	290	211	185	272	382	440	178
Mean	24.52	24.93	24.55	24.78	24.19	23.52	25.21	25.09	24.75	24.07	24.15
s.d	4.19	4.17	3.74	4.23	4.48	4.57	4.04	3.82	4.16	4.22	4.26
10th centile	19.25	19.98	19.98	19.25	19.25	17.98	20.73	19.98	19.98	19.25	19.25
15th centile	20.73	20.73	20.73	20.73	19.25	19.25	20.73	20.73	20.73	19.98	19.98
50th centile	24.11	24.11	24.11	24.11	24.11	23.21	25.03	25.03	24.11	24.11	24.11
85th centile	28.13	29.31	28.13	29.31	28.13	28.13	29.31	29.31	29.31	28.13	28.13
90th centile	29.31	30.70	29.31	30.70	29.31	29.31	30.70	29.31	30.70	29.31	30.70
**75+**											
N	1208	292	323	269	185	139	96	144	229	296	162
Mean	23.82	24.44	23.38	23.62	24.01	23.63	24.36	24.20	24.07	23.90	23.64
s.d	4.41	4.14	4.06	4.76	4.75	4.51	3.83	3.98	4.19	4.68	4.93
10th centile	29.31	19.25	18.59	17.98	18.59	17.98	29.31	29.31	29.31	29.31	30.70
15th centile	23.67	19.98	19.25	19.25	19.25	18.59	24.30	23.97	23.76	23.39	22.59
50th centile	19.25	24.11	23.21	23.21	23.21	23.21	19.98	19.98	19.25	18.59	17.43
85th centile	19.98	28.13	27.03	28.13	28.13	28.13	20.73	20.73	19.98	19.25	17.98
90th centile	23.21	29.31	29.31	29.31	29.31	30.70	24.11	24.11	23.21	23.21	22.35

	Education			Region								
	High	Other	None	North East	North West	Yorkshire and The Humber	East Midlands	West Midlands	East of England	London	South East	South West
**16-24**												
N	119	925	95	115	160	116	107	121	110	123	172	119
Mean	23.43	23.61	23.45	23.18	23.49	23.28	23.88	23.81	23.52	23.83	23.80	23.09
s.d	3.55	3.59	3.87	3.59	3.91	3.86	3.62	3.56	3.52	3.70	3.45	3.16
10th centile	19.25	19.25	17.98	18.59	17.98	18.59	19.25	19.25	18.59	19.25	19.98	19.25
15th centile	19.98	19.98	19.25	18.59	19.98	19.25	20.73	20.73	19.98	19.98	19.98	19.98
50th centile	23.21	23.21	24.11	24.11	23.21	23.21	24.11	23.21	23.21	24.11	24.11	23.21
85th centile	27.03	27.03	27.03	26.02	28.13	27.03	27.03	27.03	27.03	27.03	27.03	26.02
90th centile	28.13	28.13	28.13	27.03	28.13	27.03	28.13	28.13	28.13	28.13	28.13	27.03
**25-34**												
N	825	1310	227	108	342	245	218	208	266	369	388	222
Mean	24.19	23.17	22.47	23.58	23.50	23.17	22.81	23.70	23.74	24.17	23.48	22.83
s.d	3.41	3.60	3.65	3.40	3.50	3.63	3.37	3.69	3.36	3.82	3.48	3.30
10th centile	19.98	18.59	17.43	18.59	18.59	18.59	19.25	18.59	19.98	19.25	19.25	18.59
15th centile	20.73	19.98	18.59	19.25	19.98	19.25	19.98	19.98	20.73	20.73	19.98	19.25
50th centile	24.11	23.21	22.35	24.11	24.11	23.21	23.21	24.11	23.21	24.11	23.21	23.21
85th centile	27.03	26.02	26.02	26.02	27.03	27.03	26.02	27.03	27.03	27.03	27.03	26.02
90th centile	28.13	27.03	27.03	28.13	27.03	27.03	26.02	28.13	28.13	28.13	28.13	26.02
**35-44**												
N	707	1109	178	154	297	196	197	177	224	239	327	187
Mean	24.33	23.08	22.23	23.25	23.42	23.26	23.47	22.88	23.69	23.69	23.61	23.16
s.d	3.55	3.85	4.34	4.11	4.17	4.05	4.09	3.68	3.76	4.05	3.35	3.57
10th centile	19.98	18.59	17.43	17.98	17.98	18.59	19.25	17.98	19.25	18.59	19.25	18.59
15th centile	20.73	19.25	17.98	19.25	19.25	19.98	19.25	19.25	19.98	19.98	19.98	19.25
50th centile	24.11	23.21	22.35	23.21	23.21	23.21	23.21	23.21	23.21	24.11	23.21	23.21
85th centile	27.03	26.02	26.02	27.03	27.03	27.03	28.13	26.02	27.03	27.03	27.03	27.03
90th centile	28.13	28.13	27.03	28.13	28.13	28.13	28.13	27.03	28.13	28.13	28.13	27.03
**45-54**												
N	599	1246	259	176	311	187	188	215	234	237	335	224
Mean	24.21	23.32	22.49	22.89	23.57	23.43	23.60	22.79	23.12	24.04	23.78	23.23
s.d	3.71	3.84	4.94	4.20	4.14	3.90	3.84	4.18	3.77	4.34	3.74	3.71
10th centile	19.98	18.59	16.88	17.98	18.59	19.25	19.25	17.98	18.59	18.59	19.25	18.59
15th centile	20.73	19.25	17.98	19.25	19.25	19.98	19.98	18.59	19.25	19.98	20.73	19.25
50th centile	24.11	23.21	22.35	23.21	23.21	23.21	23.21	22.35	23.21	24.11	23.21	23.21
85th centile	27.03	27.03	27.03	27.03	27.03	27.03	27.03	26.02	26.02	28.13	27.03	27.03
90th centile	29.31	28.13	28.13	28.13	29.31	28.13	28.13	27.03	27.03	29.31	28.13	28.13
**55-64**												
N	513	1045	494	193	272	195	199	205	231	183	340	236
Mean	24.62	23.91	22.81	23.64	23.06	23.63	24.07	23.44	23.85	24.22	24.10	24.26
s.d	3.53	4.04	4.30	4.40	4.59	3.87	3.86	3.94	3.63	4.30	3.78	3.90
10th centile	19.98	19.25	17.98	18.59	17.98	18.59	19.25	19.25	19.25	19.25	19.25	19.25
15th centile	20.73	19.98	18.59	19.25	18.59	19.98	19.98	19.98	19.98	19.98	19.98	19.98
50th centile	24.11	24.11	22.35	24.11	23.21	23.21	24.11	24.11	24.11	24.11	24.11	24.11
85th centile	28.13	28.13	27.03	28.13	27.03	27.03	28.13	27.03	27.03	29.31	28.13	28.13
90th centile	29.31	29.31	28.13	29.31	28.13	28.13	28.13	28.13	28.13	29.31	28.13	29.31
**65-74**												
N	343	832	645	148	231	181	168	207	210	142	299	235
Mean	25.08	24.65	24.09	24.05	23.97	24.57	24.66	24.00	24.96	24.32	24.74	25.03
s.d	3.92	4.08	4.41	4.60	4.14	4.52	3.93	4.32	4.26	3.70	4.31	3.94
10th centile	19.98	19.98	19.25	18.59	19.25	19.25	19.98	18.59	19.25	19.25	19.98	20.73
15th centile	20.73	20.73	19.98	19.98	19.98	19.98	20.73	19.25	19.98	19.98	20.73	21.54
50th centile	25.03	24.11	24.11	23.21	24.11	24.11	24.11	24.11	25.03	24.11	24.11	25.03
85th centile	29.31	28.13	28.13	28.13	28.13	29.31	28.13	28.13	29.31	28.13	29.31	28.13
90th centile	30.70	29.31	29.31	30.70	29.31	30.70	29.31	29.31	30.70	29.31	30.70	29.31
**75+**												
N	172	478	557	93	161	107	111	129	153	98	228	128
Mean	24.30	24.26	23.30	23.64	23.52	23.86	24.64	23.50	23.65	23.04	24.22	24.05
s.d	3.50	4.31	4.69	4.86	4.21	4.48	4.75	4.23	4.50	4.26	4.31	4.48
10th centile	29.31	29.31	29.31	29.31	29.31	28.13	32.55	28.13	29.31	28.13	29.31	29.31
15th centile	24.31	23.55	23.10	23.40	23.48	23.51	23.74	23.41	23.74	23.97	23.89	23.61
50th centile	19.98	19.25	17.98	18.59	18.59	18.59	19.25	18.59	19.25	19.25	19.25	19.25
85th centile	20.73	19.98	18.59	19.25	19.25	19.25	19.98	19.25	19.98	19.98	19.98	19.98
90th centile	24.11	23.21	23.21	23.21	23.21	23.21	24.11	23.21	23.21	24.11	24.11	23.21

	Ethnicity					Self-rated health				Longstanding illness		
	White	Mixed	Asian	Black	Other	Very good	Good	Fair	Bad /Very Bad	None	Non limiting	Limiting
**16-34**												
N	2338	49	263	75	30	1306	1184	224	46	233	291	2235
Mean	23.44	23.65	23.84	25.39	23.40	24.45	23.07	21.43	19.40	23.79	23.27	21.44
s.d	3.52	2.93	3.91	4.23	3.47	3.41	3.40	3.78	3.28	3.48	3.65	3.87
10	19.25	19.98	19.25	19.98	17.43	19.98	19.25	16.88	15.32	19.25	18.59	16.88
15	19.98	21.54	20.73	20.73	18.59	21.54	19.98	17.98	15.84	19.98	19.25	17.43
50	23.21	24.11	24.11	25.03	24.11	24.11	23.21	20.73	19.98	24.11	23.21	21.54
85	27.03	27.03	27.03	30.70	27.03	27.03	26.02	25.03	23.21	27.03	27.03	25.03
90	27.03	28.13	28.13	30.70	27.03	28.13	27.03	26.02	23.21	28.13	28.13	26.02
**35-54**												
N	3604	55	277	139	27	1480	1809	579	236	745	717	2641
Mean	23.28	24.60	24.16	25.08	24.81	24.73	23.45	21.99	19.07	23.97	23.77	21.26
s.d	3.83	4.61	4.30	4.25	5.15	3.65	3.48	4.00	4.29	3.67	3.64	4.30
10	18.59	18.59	19.25	19.98	17.98	19.98	19.25	17.43	14.75	19.25	19.25	16.36
15	19.25	19.98	19.98	20.73	17.98	21.54	19.98	17.98	15.32	19.98	19.98	16.88
50	23.21	24.11	24.11	24.11	25.03	24.11	23.21	21.54	18.59	24.11	24.11	20.73
85	27.03	28.13	28.13	29.31	30.70	28.13	27.03	26.02	23.21	27.03	27.03	25.03
90	28.13	32.55	29.31	30.70	30.70	29.31	28.13	27.03	24.11	28.13	28.13	27.03
**55+**												
N	4887	15	112	46	13	1162	2105	1269	547	1952	1383	1745
Mean	24.01	23.82	24.72	25.41	25.67	25.97	24.49	22.95	20.68	24.87	24.71	22.56
s.d	4.16	4.14	4.61	4.90	4.55	3.96	3.85	3.86	3.89	4.21	3.90	4.00
10	19.25	19.25	19.25	19.98	20.73	21.54	19.98	18.59	15.84	19.98	19.98	17.43
15	19.98	19.25	19.98	20.73	23.21	22.35	20.73	19.25	16.88	20.73	20.73	18.59
50	24.11	21.54	24.11	24.11	25.03	25.03	24.11	22.35	20.73	25.03	24.11	22.35
85	28.13	29.31	29.31	30.70	29.31	29.31	28.13	27.03	25.03	29.31	28.13	27.03
90	29.31	29.31	32.55	35.00	35.00	30.70	29.31	28.13	25.03	30.70	29.31	28.13

S3. SWEMWBS scores for women by age group

		Index of Multiple Deprivation					Income quintiles				
	ALL	Least	2nd	3rd	4th	Most	Highest	2nd	3rd	4th	Lowest
**16-24**											
N	1540	259	301	312	303	365	122	246	213	245	360
Mean	23.17	23.07	23.47	23.27	23.44	22.62	23.81	23.40	22.94	22.81	22.69
s.d	3.86	3.77	3.54	3.97	4.15	3.80	4.08	3.56	3.61	3.76	4.15
10th centile	18.59	17.98	19.25	18.59	18.59	17.98	18.59	19.25	18.59	17.98	17.98
15th centile	19.25	19.25	19.98	19.25	19.25	18.59	19.98	19.98	19.25	19.25	18.59
50th centile	23.21	23.21	23.21	23.21	23.21	23.21	24.11	23.21	23.21	22.35	22.35
85th centile	27.03	27.03	27.03	27.03	27.03	26.02	27.03	26.02	27.03	27.03	27.03
90th centile	28.13	28.13	28.13	28.13	28.13	27.03	29.31	28.13	28.13	28.13	27.03
**25-34**											
N	2282	334	387	501	502	558	565	496	366	380	337
Mean	23.68	24.18	23.83	23.65	23.56	23.40	24.54	23.96	23.69	23.25	22.46
s.d	3.80	3.56	3.62	3.80	3.78	4.04	3.23	3.51	3.62	3.88	4.51
10th centile	19.25	19.98	19.25	19.25	19.25	18.59	20.73	19.25	19.25	18.59	17.43
15th centile	19.98	20.73	19.98	19.98	19.98	19.25	21.54	19.98	20.73	19.25	17.98
50th centile	24.11	24.11	24.11	24.11	23.21	23.21	24.11	24.11	23.21	23.21	22.35
85th centile	27.03	27.03	27.03	27.03	27.03	27.03	27.03	27.03	27.03	27.03	26.02
90th centile	28.13	28.13	28.13	28.13	28.13	28.13	28.13	28.13	28.13	28.13	28.13
35-44											
N	2682	636	552	547	495	452	604	549	401	407	365
Mean	23.46	23.84	23.75	23.56	23.25	22.69	24.23	23.82	23.52	22.59	22.41
s.d	3.89	3.61	3.55	3.98	3.79	4.51	3.70	3.68	3.67	3.83	4.18
10th centile	18.59	19.25	19.25	19.25	18.59	17.43	19.98	19.25	19.25	17.98	17.43
15th centile	19.25	19.98	19.98	19.25	19.25	17.98	20.73	19.98	19.98	18.59	17.98
50th centile	23.21	24.11	23.21	23.21	23.21	22.35	24.11	23.21	23.21	22.35	22.35
85th centile	27.03	27.03	27.03	27.03	27.03	27.03	28.13	27.03	27.03	26.02	27.03
90th centile	28.13	28.13	28.13	28.13	28.13	28.13	29.31	28.13	28.13	27.03	28.13
**45-54**											
N	2840	703	581	582	529	445	626	596	442	365	393
Mean	23.31	23.95	23.67	23.13	23.07	22.31	24.29	23.90	23.17	22.73	21.39
s.d	3.88	3.57	3.68	3.61	3.97	4.57	3.56	3.40	3.93	3.84	4.32
10th centile	18.59	19.25	19.25	18.59	18.59	17.43	19.98	19.25	18.59	18.59	16.36
15th centile	19.25	20.73	19.98	19.25	19.25	18.59	20.73	19.98	19.25	19.25	17.43
50th centile	23.21	24.11	24.11	23.21	23.21	22.35	24.11	24.11	23.21	22.35	21.54
85th centile	27.03	27.03	27.03	26.02	27.03	26.02	28.13	27.03	27.03	26.02	25.03
90th centile	28.13	28.13	28.13	27.03	28.13	28.13	29.31	28.13	28.13	28.13	26.02
**55-64**											
N	2431	604	584	532	397	314	418	530	428	352	276
Mean	23.95	24.17	24.20	24.26	23.72	22.73	24.49	24.39	24.08	23.79	22.54
s.d	4.10	3.87	3.96	4.12	4.06	4.58	3.89	3.86	3.93	4.19	4.50
10th centile	19.25	19.25	19.25	19.25	18.59	16.88	19.25	19.98	19.25	18.59	16.88
15th centile	19.98	19.98	20.73	19.98	19.98	17.98	20.73	20.73	19.98	19.98	17.98
50th centile	24.11	24.11	24.11	24.11	23.21	22.35	24.11	24.11	24.11	23.21	22.35
85th centile	28.13	27.03	28.13	28.13	28.13	27.03	28.13	28.13	28.13	28.13	27.03
90th centile	29.31	29.31	29.31	29.31	28.13	29.31	29.31	29.31	29.31	29.31	28.13
**65-74**											
N	1964	477	475	435	334	243	160	226	422	444	276
Mean	24.26	24.55	24.40	24.48	23.53	23.98	24.71	25.16	24.52	23.91	23.76
s.d.	4.31	4.24	4.34	4.33	4.07	4.61	4.66	3.90	4.12	4.16	4.47
10th centile	19.25	19.25	19.25	19.98	18.59	18.59	19.25	20.73	19.25	19.25	18.59
15th centile	19.98	19.98	19.98	19.98	19.98	19.25	20.73	21.54	19.98	19.98	19.98
50th centile	24.11	24.11	24.11	24.11	24.11	23.21	24.11	25.03	24.11	23.21	23.21
85th centile	28.13	29.31	28.13	29.31	27.03	29.31	29.31	29.31	28.13	28.13	28.13
90th centile	30.70	29.31	30.70	30.70	29.31	30.70	30.70	30.70	30.70	29.31	29.31
**75+**											
N	1482	335	376	289	268	214	74	108	255	359	262
Mean	23.59	23.86	23.82	23.83	22.90	23.33	24.21	24.15	23.52	23.73	23.53
s.d	4.23	4.20	4.17	4.36	4.40	4.35	3.77	4.02	4.09	4.55	4.27
10th centile	18.59	18.59	19.25	18.59	17.98	17.98	29.31	29.31	29.31	29.31	29.31
15th centile	19.25	19.98	19.25	19.25	19.25	18.59	24.35	24.02	23.64	23.24	22.59
50th centile	23.21	24.11	23.21	23.21	22.35	23.21	19.98	19.98	19.25	18.59	17.43
85th centile	28.13	28.13	28.13	28.13	27.03	28.13	20.73	20.73	19.98	19.25	18.59
90th centile	29.31	29.31	29.31	29.31	28.13	29.31	24.11	24.11	23.21	23.21	22.35

	Education			Region								
	High	Other	None	North East	North West	Yorkshire and The	East Midlands	West Midlands	East of England	London	South East	South West
**16-24**												
N	202	1214	121	132	206	148	145	154	158	195	252	150
Mean	24.65	22.94	22.84	22.53	23.14	22.53	23.80	23.12	22.72	23.72	22.93	23.59
s.d	3.68	3.79	4.31	4.09	3.73	3.85	3.90	3.62	3.81	4.14	3.62	3.94
10	20.73	18.59	17.98	17.43	17.98	17.98	18.59	19.25	17.98	18.59	18.59	18.59
15	20.73	19.25	17.98	17.98	19.25	18.59	19.25	19.98	18.59	19.98	19.25	19.25
50	24.11	23.21	23.21	23.21	23.21	22.35	24.11	23.21	23.21	24.11	23.21	23.21
85	28.13	26.02	27.03	26.02	26.02	26.02	28.13	26.02	27.03	28.13	26.02	28.13
90	29.31	27.03	28.13	27.03	28.13	27.03	28.13	27.03	28.13	29.31	27.03	29.31
**25-34**												
N	854	1438	196	125	314	254	234	218	294	407	414	232
Mean	24.12	23.28	23.41	23.04	24.03	23.74	23.41	23.21	23.77	23.91	23.93	23.25
s.d	3.50	3.91	4.50	3.65	4.06	3.98	3.96	3.27	3.85	3.92	3.54	3.63
10	19.98	18.59	18.59	18.59	19.25	19.25	19.25	19.25	19.25	19.25	19.25	18.59
15	20.73	19.25	19.25	19.25	19.98	19.98	19.25	19.98	19.98	19.98	20.73	19.98
50	24.11	23.21	23.21	23.21	24.11	24.11	23.21	23.21	24.11	24.11	24.11	23.21
85	27.03	27.03	27.03	27.03	27.03	27.03	27.03	26.02	27.03	28.13	27.03	26.02
90	28.13	28.13	29.31	27.03	29.31	28.13	28.13	27.03	28.13	29.31	28.13	27.03
35-44												
N	943	1532	202	233	335	255	265	226	305	361	454	248
Mean	24.21	23.18	22.18	22.82	23.52	23.27	23.12	23.25	23.64	23.91	23.57	23.22
s.d	3.62	3.94	4.07	3.74	4.39	3.69	4.07	3.76	4.04	3.79	3.76	3.56
10	19.98	18.59	17.43	17.98	18.59	18.59	18.59	18.59	19.25	19.25	19.25	18.59
15	20.73	19.25	17.98	19.25	19.25	19.98	19.25	19.25	19.98	19.98	19.98	19.25
50	24.11	23.21	22.35	23.21	23.21	23.21	23.21	23.21	23.21	24.11	23.21	23.21
85	27.03	27.03	26.02	26.02	27.03	27.03	27.03	27.03	27.03	27.03	27.03	27.03
90	28.13	28.13	27.03	27.03	28.13	27.03	28.13	28.13	28.13	28.13	28.13	28.13
**45-54**												
N	755.00	1772.00	310.00	248.00	382.00	272.00	258.00	303.00	305.00	306.00	472.00	294.00
Mean	24.14	23.18	22.02	22.51	23.09	23.54	23.01	23.22	23.47	23.64	23.45	23.25
s.d	3.68	3.74	4.66	3.89	4.09	4.08	3.78	4.06	3.75	3.94	3.74	3.53
10	19.98	18.59	16.88	17.98	18.59	19.25	18.59	18.59	19.25	19.25	19.25	18.59
15	20.73	19.25	17.98	18.59	19.25	19.98	19.25	19.25	19.98	19.98	19.25	19.25
50	24.11	23.21	22.35	22.35	23.21	23.21	23.21	23.21	23.21	23.21	23.21	23.21
85	27.03	27.03	26.02	26.02	27.03	28.13	26.02	27.03	27.03	27.03	27.03	27.03
90	29.31	28.13	27.03	27.03	28.13	28.13	27.03	28.13	28.13	29.31	28.13	27.03
**55-64**												
N	438	1366	627	235	295	250	218	233	281	200	419	300
Mean	24.55	24.04	23.31	23.41	24.22	23.59	23.67	23.38	24.19	23.68	24.37	24.26
s.d	3.69	4.03	4.43	3.77	4.18	4.45	3.87	4.17	4.30	3.99	4.10	3.73
10	19.98	19.25	17.98	18.59	19.25	17.98	19.25	18.59	19.25	18.59	19.98	19.25
15	20.73	19.98	19.25	19.98	20.73	19.25	19.98	19.25	19.98	19.98	20.73	19.98
50	24.11	24.11	23.21	23.21	24.11	23.21	24.11	23.21	24.11	23.21	24.11	24.11
85	28.13	28.13	28.13	27.03	28.13	28.13	27.03	27.03	29.31	28.13	28.13	27.03
90	29.31	29.31	29.31	28.13	29.31	29.31	28.13	28.13	30.70	28.13	29.31	28.13
**65-74**												
N	220	875	868	161	265	179	206	202	222	157	349	223
Mean	24.90	24.67	23.67	23.81	23.93	24.10	24.11	23.97	24.54	23.93	24.68	24.68
s.d	3.96	4.20	4.44	4.35	4.37	4.61	4.29	4.28	3.93	4.27	4.20	4.57
10	19.98	19.98	18.59	18.59	18.59	19.25	19.25	18.59	20.73	19.25	19.98	19.25
15	20.73	20.73	19.25	19.25	19.98	19.98	19.98	19.98	20.73	19.98	20.73	20.73
50	25.03	24.11	23.21	23.21	24.11	24.11	24.11	24.11	24.11	23.21	24.11	24.11
85	28.13	29.31	28.13	28.13	28.13	29.31	28.13	28.13	28.13	29.31	29.31	29.31
90	29.31	30.70	29.31	30.70	29.31	30.70	29.31	29.31	29.31	29.31	30.70	32.55
**75+**												
N	72	496	913	123	199	147	136	157	164	144	238	174
Mean	23.35	24.36	23.21	23.62	23.18	23.65	23.22	23.38	23.67	23.53	24.28	23.58
s.d	3.27	4.28	4.22	4.27	4.22	4.10	3.86	4.37	4.40	4.47	4.45	3.74
10	29.31	30.70	29.31	29.31	29.31	29.31	28.13	29.31	29.31	30.70	30.70	28.13
15	24.26	23.46	23.15	23.03	23.58	23.46	23.45	23.33	23.70	23.79	23.81	23.67
50	19.98	18.59	17.98	17.98	18.59	18.59	18.59	18.59	19.25	19.25	19.25	19.25
85	20.73	19.25	18.59	19.25	19.25	19.25	19.25	19.25	19.98	19.98	19.98	19.98
90	24.11	23.21	23.21	23.21	23.21	23.21	23.21	23.21	23.21	23.21	24.11	23.21

	Ethnicity					Self-rated health				Longstanding illness		
						Very			Bad/Very		Non	
	White	Mixed	Asian	Black	Other	good	Good	Fair	Bad	None	limiting	Limiting
**16-34**												
N	3183	83	374	126	45	1533	1734	457	98	2918	417	484
Mean	23.35	23.24	24.07	23.91	23.22	24.48	23.26	21.22	20.10	23.76	23.20	21.59
s.d	3.72	4.24	4.41	4.06	4.12	3.73	3.54	3.83	3.93	3.76	3.51	4.04
10	18.59	17.98	18.59	19.25	18.59	19.98	18.59	16.88	15.84	19.25	19.25	16.88
15	19.25	19.25	19.98	19.98	19.25	20.73	19.98	17.98	16.36	19.98	19.98	17.43
50	23.21	23.21	24.11	24.11	22.35	24.11	23.21	20.73	19.98	24.11	23.21	21.54
85	27.03	28.13	28.13	28.13	26.02	28.13	26.02	25.03	23.21	27.03	26.02	25.03
90	28.13	28.13	29.31	29.31	28.13	29.31	27.03	26.02	25.03	28.13	27.03	27.03
**35-54**												
N	#####	57.23	316.41	166.27	40.49	1866.35	2077.34	710.70	274.12	3139.29	816.29	971.34
Mean	23.32	23.41	23.84	24.34	23.34	24.71	23.41	21.39	19.32	23.98	23.64	21.25
s.d	3.82	4.19	4.25	4.36	4.72	3.60	3.46	3.72	4.28	3.59	3.74	4.18
10	18.59	18.59	19.25	19.25	17.98	20.73	19.25	17.43	14.75	19.25	19.25	16.36
15	19.25	19.25	19.98	19.98	18.59	21.54	19.98	17.98	15.32	20.73	19.98	17.43
50	23.21	23.21	23.21	24.11	23.21	24.11	23.21	20.73	19.25	24.11	23.21	20.73
85	27.03	27.03	28.13	29.31	28.13	28.13	27.03	25.03	23.21	27.03	27.03	25.03
90	28.13	28.13	29.31	29.31	29.31	29.31	27.03	26.02	25.03	28.13	28.13	26.02
**55+**												
N	4581	16	97	51	13	1139	1961	1224	440	1828	1108	1829
Mean	23.96	24.73	23.53	23.97	24.24	25.77	24.33	22.84	20.68	24.88	24.65	22.61
s.d	4.19	3.28	4.52	5.29	4.29	4.07	3.92	3.82	4.03	4.13	3.87	4.13
10	19.25	20.73	17.98	17.43	18.59	20.73	19.98	18.59	16.36	19.98	19.98	17.98
15	19.98	21.54	18.59	18.59	21.54	21.54	20.73	19.25	16.88	20.73	20.73	18.59
50	24.11	24.11	23.21	24.11	25.03	25.03	24.11	22.35	19.98	25.03	24.11	22.35
85	28.13	28.13	28.13	29.31	26.02	30.70	28.13	27.03	25.03	29.31	28.13	27.03
90	29.31	28.13	29.31	32.55	27.03	30.70	29.31	28.13	25.03	30.70	30.70	28.13

S4 Spearman correlation coefficient between SWEMWBS(N=13584)/WEMWBS (N=13585) and health variables across split samples, HSE 2010-13

Split sample

	SWEMWBS (N1)		WEMWBS (N2)	
	N1	ρ	N2	ρ
WEMWBS/SWEMWBS	13338	0.95***	13279	0.97***
Self-rated health	13583	-0.33***	13276	0.36***
Limiting longstanding illness	13576	-0.21***	13272	-0.23***
GHQ12	5887	-0.52***	5630	-0.53***
Happiness scale	6481	0.54***	6318	0.57***
EQ-5D self-reported health state	8986	0.40***	8758	0.42***

S5. Logistic regression establishing if SWEMWBS (N=15910) categorised into low (7-19.25), medium (19.98-27.03) and high (28.13-35) replicates associations with WEMWBS (N=15,652) categorised HSE 2010,2011,2013 listwise deletion

	Low verus medium		High versus medium	
	**SWEMWBS**	**WEMWBS**	**SWEMWBS**	**WEMWBS**
Variables (reference category)	OR (95% CI)	OR (95% CI)	OR (95% CI)	OR (95% CI)
**Sex (Men)**	1	1	1	1
Women	1.02(0.92-1.12)	1.08(0.97-1.20)	1.07(0.96-1.18)	1.03(0.93-1.14)
**Age group (16-24)**	1	1	1	1
25-34	**1.24(0.99-1.57)**	**1.25(0.98-1.58)**	0.90(0.68-1.19)	0.82(0.62-1.07)
35-44	**1.46(1.17-1.84)**	**1.65(1.31-2.09)**	1.01(0.77-1.32)	0.90(0.69-1.17)
45-54	**1.33(1.05-1.67)**	**1.55(1.22-1.97)**	1.03(0.78-1.36)	0.95(0.72-1.25)
55-64	0.89(0.69-1.16)	0.97(0.74-1.27)	**1.59(1.18-2.13)**	**1.40(1.04-1.88)**
65-74	**0.64(0.46-0.90)**	0.71(0.50-1.01)	**2.14(1.52-3.01)**	**1.97(1.40-2.76)**
75+	0.75(0.52-1.08)	0.74(0.51-1.09)	**2.19(1.52-3.17)**	**2.00(1.39-2.88)**
**General Health (Very good)**	1	1	1	1
Good	**1.73(1.49-2.00)**	**1.83(1.56-2.14)**	**0.54(0.48-0.61)**	**0.53(0.47-0.59)**
Fair	**4.20(3.54-4.97)**	**4.97(4.15-5.96)**	**0.31(0.26-0.38)**	**0.31(0.25-0.37)**
Bad /Very Bad	**8.98(7.19-11.23)**	**12.29(9.81-15.40)**	**0.17(0.11-0.27)**	**0.17(0.11-0.27)**
**Marital Status (Single)**	1	1	1	1
Maried/cohabitees	**0.72(0.62-0.83)**	**0.64(0.55-0.75)**	**1.11(0.93-1.33)**	1.09(0.90-1.31)
Seperated/widowed/divorced	0.88(0.73-1.07)	0.87(0.72-1.07)	1.01(0.81-1.27)	0.97(0.77-1.23)
**Ethnic group (White)**	1	1	1	1
Mixed	0.73(0.43-1.23)	**0.44(0.23-0.84)**	1.11(0.67-1.84)	0.92(0.55-1.53)
Asian	0.83(0.63-1.08)	0.84(0.64-1.10)	**1.46(1.13-1.89)**	**1.55(1.20-2.00)**
Black	**0.52(0.33-0.81)**	0.57(0.36-0.91)	**2.94(2.15-4.02)**	**3.14(2.29-4.31)**
Other	0.72(0.34-1.52)	0.85(0.40-1.83)	1.27(0.67-2.40)	1.61(0.93-2.79)
**Education (Degree or higher)**	1	1	1	1
Below Degree	**1.22(1.05-1.42)**	**1.22(1.04-1.42)**	0.95(0.84-1.08)	0.95(0.84-1.08)
No Qualification	**1.43(1.16-1.75)**	**1.36(1.11-1.67)**	1.04(0.87-1.25)	1.06(0.89-1.27)
**Economic Activity (In employmen**	1	1	1	1
ILO unemployed	**1.38(1.09-1.74)**	**1.21(0.94-1.56)**	1.00(0.74-1.34)	1.24(0.95-1.61)
Retired	1.06(0.84-1.34)	1.04(0.82-1.32)	**1.23(1.01-1.49)**	**1.18(0.96-1.44)**
Other economically inactive	**1.66(1.41-1.94)**	**1.66(1.40-1.95)**	1.14(0.95-1.38)	1.12(0.93-1.36)
**Equivalised Income Quintile (High**	1	1	1	1
2nd	1.06(0.88-1.27)	1.02(0.85-1.23)	0.93(0.79-1.09)	0.90(0.77-1.05)
3rd	**1.22(1.01-1.47)**	1.14(0.94-1.37)	0.89(0.75-1.06)	0.85(0.72-1.01)
4th	**1.33(1.09-1.62)**	**1.28(1.05-1.56)**	**0.80(0.66-0.97)**	**0.80(0.66-0.97)**
Lowest	**1.42(1.16-1.73)**	**1.42(1.16-1.74)**	0.89(0.72-1.10)	0.90(0.72-1.12)
**Body Mass Index (Normal)**	1	1	1	
Underweight	1.44(0.98-2.12)	1.51(1.01-2.24)	0.83(0.50-1.36)	0.99(0.59-1.66)
Overweight	0.96(0.84-1.10)	1.00(0.87-1.15)	1.07(0.94-1.22)	1.02(0.90-1.16)
Obese	0.95(0.82-1.10)	0.97(0.84-1.13)	**1.25(1.08-1.45)**	**1.24(1.06-1.44)**
Morbidly obese	0.98(0.74-1.30)	1.03(0.77-1.37)	**1.78(1.30-2.44)**	**1.49(1.07-2.08)**
**Fruit and Vegetable intake (5 or more portions/day)**	1	1	1	1
3 to <5 portions/day	0.95(0.82-1.10)	0.97(0.84-1.13)	**0.85(0.75-0.96)**	**0.87(0.76-0.98)**
1 to <3 portions/day	1.08(0.93-1.24)	1.14(0.99-1.32)	**0.79(0.69-0.92)**	**0.75(0.65-0.87)**
<1 portions/day	**1.37(1.14-1.65)**	**1.50(1.24-1.82)**	0.82(0.65-1.04)	0.83(0.66-1.05)
**Alcohol drinking (Non-drinker)**	1	1	1	1
Moderate	0.90(0.79-1.04)	**0.85(0.73-0.98)**	**0.83(0.73-0.94)**	**0.84(0.74-0.95)**
Excess	0.96(0.81-1.13)	0.96(0.81-1.14)	0.87(0.75-1.02)	0.92(0.78-1.08)
Heavy episodic	0.99(0.85-1.16)	0.97(0.83-1.14)	**0.79(0.67-0.93)**	**0.81(0.68-0.95)**
**Smoking**	1	1	1	1
Ex smoker	1.06(0.93-1.20)	1.07(0.94-1.22)	0.97(0.86-1.09)	0.96(0.85-1.08)
Current smoker	**1.33(1.15-1.52)**	**1.25(1.09-1.45)**	1.04(0.89-1.22)	0.97(0.83-1.14)
